# Tracing Mercury from Land to River: Global Sources, Retention, and Implications for Sustainability

**DOI:** 10.1021/acs.est.6c03367

**Published:** 2026-07-25

**Authors:** Dong Peng, Zeli Tan, Peipei Wu, Ruirong Chang, Shaojian Huang, Peng Zhang, Yujuan Wang, Zhengcheng Song, Yanxu Zhang, Ting Lei, Maodian Liu, Beth A. Middleton, Jianhua Gao, Junguo Liu, Guangchun Lei, Shu Tao

**Affiliations:** † School of Geography and Ocean Science, Ministry of Education Key Laboratory for Coast and Island Development, 12581 Nanjing University, Nanjing 210023, China; ‡ School of Atmospheric Sciences, Nanjing University, Nanjing 210023, China; § 6865 Pacific Northwest National Laboratory, Richland, Washington 99354, United States; ∥ Scripps Institution of Oceanography, University of California San Diego, La Jolla, California 92093, United States; ⊥ Department of Earth and Environmental Sciences, Tulane University, New Orleans, Louisiana 70118, United States; # School of Ecology and Nature Conservation, 12380 Beijing Forestry University, Beijing 100083, China; ∇ College of Urban and Environmental Sciences, 12465 Peking University, Beijing 100871, China; ○ U.S. Geological Survey, Wetland and Aquatic Research Center, Lafayette, Louisiana 70506, United States; ◆ Henan Provincial Key Laboratory of Hydrosphere and Watershed Water Security, North China University of Water Resources and Electric Power, Zhengzhou 450045, China; ¶ School of Environmental Science and Engineering, 255310 Southern University of Science and Technology, Shenzhen 518055, China

**Keywords:** mercury, riverine transport, anthropogenic mercury release, earth system model, water management, pollution governance

## Abstract

Mercury (Hg) pollution in river systems is a global sustainability challenge. Yet the transport, transformation, and retention of Hg within global rivers remain poorly quantified, particularly in regions with sparse observations such as Southeast Asia and Africa, hindering effective pollution mitigation and reinforcing geographic inequities in scientific knowledge and environmental governance. Here, we present the first global, high-resolution simulation of riverine Hg dynamics using a process-based model that traces Hg from land-based sources through river networks to the ocean. Under a realistic scenario, we estimate that ∼1900 megagrams per year (Mg/yr) of Hg enters global rivers, including 1500 Mg/yr from human-induced sources and 400 Mg/yr from soil erosion. Nearly half of this flux (∼1000 Mg/yr) is retained in reservoirs and dams, which act as major sinks. While such retention limits downstream delivery to the oceans, it also heightens in-reservoir Hg methylation risks. By bridging the gap between Hg releases and observed riverine exports, our framework offers a scalable tool for data-limited regions, promotes data access, and supports global freshwater and pollution-management strategies.

## Graphical abstract

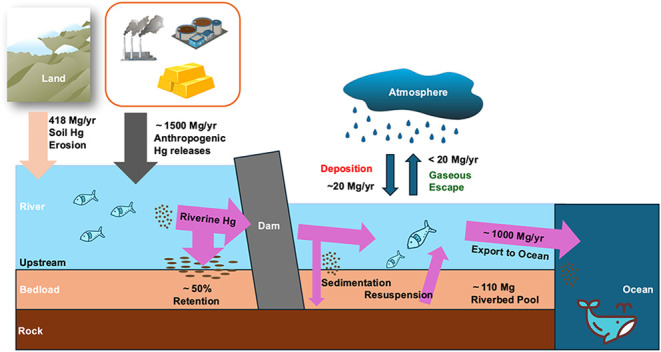

## 1 Introduction

Mercury (Hg) is one of the most toxic and persistent pollutants affecting river systems, posing serious threats to water quality, aquatic ecosystems, and human health worldwide.^1–4^ As a key component of the global Hg cycle, rivers serve as critical conduits linking terrestrial Hg sources with oceanic reservoirs.^1^ Extensive discharges of Hg-contaminated wastewater and industrial waste have elevated Hg concentrations in freshwater systems, leading to widespread bioaccumulation in aquatic organisms and increasing exposure risks for human populations.^5–7^ These impacts undermine the capacity of rivers to provide clean water and safe food, particularly in communities reliant on freshwater fisheries.^8^ To address this problem, regulatory measures such as the U.S. Clean Water Act and national fish consumption advisories have been introduced to mitigate riverine Hg pollution and its impacts.^9,10^ At the international level, the Minamata Convention on Mercury (referred to as Minamata Convention) seeks to reduce anthropogenic Hg emissions and releases globally. Despite decades of localized monitoring efforts,^11,12^ our understanding of the global-scale sources, transport pathways, and retention processes of riverine Hg remains fragmented. This limits the ability to design effective mitigation strategies and assess the progress toward sustainability goals.

Riverine Hg originates from various natural and anthropogenic sources, including natural and legacy Hg releases by soil erosion, industrial wastewater (e.g., metal smelting and/or chlor-alkali production), and artisanal and small-scale gold mining (ASGM).^13^ At present, anthropogenic releases are the primary contributors to global riverine Hg.^14^ However, there are relatively large uncertainties associated with the existing release inventories of rivers. For example, Kocman et al.^14^ estimated that approximately 1000 Mg (300–1300 Mg) of anthropogenic Hg is discharged into global water systems annually, with contributions of 220 Mg from point sources, 40 Mg from the remobilization of contaminated systems, and 440 Mg from ASGM. In another study, Streets et al.^15^ estimated that the amount of global Hg released to land and water reached up to 7280 Mg by 2010, but noted the challenge in separating releases between land and water due to a lack of data. Anthropogenic Hg emissions and releases have shifted from developed regions to developing regions,^5,16^ where ASGM and fuel combustion remain high even after the Minamata Convention.^13^ These developing regions often have less stringent environmental regulations and limited Hg monitoring in river systems, potentially increasing the risk of Hg exposure for both humans and wildlife. Consequently, a process-based modeling approach can help to address these gaps and promote more equitable Hg governance.

Riverine Hg primarily exists in the form of particulate Hg within suspended particulate matter (SPM) in rivers.^17,18^ Thus, the riverine Hg is largely influenced by sediment flux.^1,2^ At the global scale, the majority of riverine Hg discharge is in the particulate phase.^1,19^ Natural processes, especially soil erosion, are important contributors to riverine Hg concentrations and fluxes,^20^ and they influence Hg concentrations in SPM in rivers.^2,21^ Human activities, particularly those related to water management practices (e.g., the construction of reservoirs and dams worldwide), profoundly impact the processes governing riverine Hg transport in contemporary times. Reservoirs have substantial efficacy in trapping SPM,^22–24^ leading to the potential accumulation of Hg-laden sediments behind dams. For example, the Three Gorges Dam in China has resulted in the settling of 100–200 Mg of riverine Hg in bedload^2^ and has reduced the downstream flux of riverine Hg by more than 70%.^20^ However, the overall impact of reservoirs and dams on river Hg transport on a global scale remains poorly understood.

In this study, we develop a process-based global Hg model to simulate the contemporary transport and fate of Hg in global river systems. While previous work^25^ quantified long-term anthropogenic perturbations on coastal Hg export via preindustrial baselines, it lacked dynamic simulations of contemporary anthropogenic Hg processes within land-river systems. This study shifts the focus to modern temporal scales to investigate active biogeochemical mechanisms. By explicitly coupling a water management^26^ with dynamic in-stream sediment routing, we resolve certain components of the process, including contemporary terrestrial erosion, dam trapping, in-channel sedimentation, and resuspension. Note that the term “trapping” hereafter refers to the retention of materials within reservoirs or dams as simulated by the water management module. This structural advancement explicitly partitions transient riverbed sinks from permanent reservoir retention. Furthermore, using scenario-based perturbations of anthropogenic Hg releases, we systematically quantify active Hg inventories and assess how localized inputs propagate through the global watershed.

We hypothesize that (1) river routing and water infrastructure reshape the magnitude and composition of Hg delivered to oceans, (2) human-induced terrestrial Hg releases dominate present-day riverine Hg transport, and (3) reservoir trapping produces geographically uneven Hg retention, altering the coastal accumulation patterns. The study aims to (1) model present-day riverine Hg transport and export fluxes, including human-induced releases and the effects of reservoirs and dams, (2) quantify key processes controlling Hg transport to downstream sinks, and (3) estimate the global Hg accumulation within reservoirs. Here, riverine transport flux refers to Hg fluxes conveyed within river channels, whereas riverine export flux denotes the Hg flux ultimately delivered by rivers to the ocean. By resolving inland rivers and reservoir retention globally, our framework provides a scalable tool for data-limited regions, informs more equitable Hg pollution and exposure management, and enables the assessment of freshwater food safety, particularly in low-income regions. In doing so, this work can inform efforts to improve freshwater sustainability and access, particularly by clarifying how contaminant transport affects the riverine water quality.

## 2 Methods

### 2.1 Model Platform

In this study, we introduce the model for scale-adaptive river transport for mercury under water management (MOSART-Hg-WM) to simulate Hg cycling in land-river systems. This framework builds upon our previous MOSART-Hg model^25^ by integrating a water management module to represent reservoir dynamics.^26,27^ MOSART-Hg-WM is implemented within the Community Earth System Model version 2.1.3 (CESM2.1.3). It incorporates advanced physical modules originally developed for the Energy Exascale Earth System Model (E3SM), specifically coupling the core Hg cycling processes with modules for sediment transport,^28^ dam trapping,^26^ and land erosion.^29,30^

The physical hydro-sedimentological performance of this model architecture, including soil erosion, runoff, and dam trapping, has been thoroughly validated in prior studies.^26,28–32^ In this paper, we use the same present-day land-river Hg framework applied in Peng et al.,^25^ including the validated water management module for dam trapping, and refer to this configuration as MOSART-Hg-WM to distinguish it from the preindustrial configuration without modern dam regulation (MOSART-Hg). The underlying Hg biogeochemical processes remain unchanged from the previously established MOSART-Hg framework. The water management module was originally developed within E3SM,^26^ and is implemented here in CESM-MOSART-Hg to simulate Hg trapping by dams. Regarding fixed riverine boundary conditions, the model incorporates soil erosion as the primary input vector for natural and legacy Hg, while explicitly accounting for contemporary anthropogenic releases. Here, legacy Hg refers to the elevation of soil Hg concentrations relative to the ∼1850 baseline (natural background), arising from the cumulative accumulation of Hg via atmospheric deposition and other input pathways. Erosional Hg fluxes are calculated as the product of the eroded sediment flux and the corresponding soil or sediment Hg concentration. The detailed mechanistic processes governing this mobilization are described in previous work.^25^ Furthermore, while atmospheric Hg can enter rivers directly via precipitation, this model focuses exclusively on indirect loading via watershed runoff and soil erosion. Direct atmospheric deposition to the water surface is considered negligible in this framework due to the minimal surface area of the river network relative to the total catchment area.^33^

Since the water management module has been integrated, the model now accounts for global reservoir construction, determining reservoir trapping efficiency.^22,26,27^ The model facilitates the determination of trapping efficiency by leveraging input data sets of global dams through the calculation method outlined by Vörösmarty et al.^22^ Consequently, the sediment trapping processes can be effectively ascertained. This methodology finds broad application in hydrology and biogeochemical models, exemplified by its utilization in models such as the Global Nutrient Export from Watersheds 2 (NEWS 2).^34^ Data on global reservoirs are sourced from the Global Reservoir and Dam (GRanD) database,^35^ initialized on a grid basis by Zhou et al.,^26^ and originally implemented in E3SM simulations. This data set encompasses dam locations, reservoir capacities, and major functions for over 4200 dams worldwide. The trapping efficiency is calculated as follows^22,28^

1 $$e_{\mathrm{trap}}=1-\frac{0.05}{\Delta t_{\mathrm{local}}^{0.05}}$$

where Δ*t*_local_^0.05^ is the increase of local water residence time due to the reservoir [years], estimated as the effective reservoir storage capacity divided by the mean annual inflow from the reservoir upstream.

The deposition of particulate Hg to the riverbed is governed by the MOSART sediment routing scheme, occurring dynamically when local flow velocities decrease in low-gradient reaches and the suspended particulate load exceeds the flow transport capacity. Because the model operates at 0.5° or 1° spatial resolution, rivers and reservoirs are represented as subgrid parameters rather than explicitly resolved spatial polygons. Consequently, direct surface area ratios between rivers and reservoirs are not defined. Reservoir trapping of Hg is instead mathematically parametrized (eq 1), utilizing subgrid flow dynamics and water residence times to calculate retention efficiency.

The simulations span the period 1995–2004, with the initial five years used to establish the steady state of the simulated river systems (i.e., spin-up), and the subsequent five years are considered for analysis. The spatial resolution matches that of previous studies, with the Community Land Model 5.0 for Mercury (CLM5-Hg),^25^ and MOSART-Hg-WM running at resolutions of 0.9° × 1.25° and 0.5° × 0.5°, respectively. The former has been extensively evaluated in simulating erosional Hg dynamics.^25^ We include global soil Hg data sets from Wang et al.^36^ and Liu et al.^37^ to represent its uncertainties (Table 1). Furthermore, the model explicitly simulates the in-stream sedimentation of Hg.

**Table 1:** Experimental Design

experiment ID	experiment condition	soil Hg concentration data set	anthropogenic Hg release (Mg/yr)	release scenario^a^	soil Hg dynamic	atmospheric Hg deposition	water management (reservoir)	spatial distribution of release	LULCC dynamics
**Baseline**		Wang (data set)	∼1500	*Revised AMAP*	Constant	No	Yes	AMAP 2015	*LUH2*
**Anthropogenic release uncertainty**			0	*No*					
			1100	*Low*					
			1500	*Moderate*					
			2000	*High ASGM*					
			2000	*High Industrial*					
**Reservoir**	No Release without Reservoir (R)		0	*No*			No		
	No Release with R						Yes		
	Baseline without R		∼1500	*Revised AMAP*			No		
	Baseline with R						Yes		
**Inventory**	AMAP		1500	*Moderate*			Yes	AMAP 2015	
	Streets							Streets	
**Soil Hg**	Wang	Wang						AMAP 2015	
	Liu	Liu							
**Deposition**	Deposition	Wang			Dynamic	Yes			
	Without Deposition				Constant	No			

a The details of the release scenarios are given in Table 2. LULCC represents land use and land cover change, Wang represents the soil Hg data set from Wang et al.,^36^ Liu represents the soil Hg concentration from Liu et al.,^37^ AMAP 2015 represents the inventory by Steenhuisen and Wilson,^42^ Steenhuisen and Wilson,^47^ and Streets represents the inventory of Streets et al.^43^ LUH2 represents the land use and land cover data sets of Land Use Harmonization 2.

Climate forcing data, obtained from the Global Soil Wetness Project Phase 3 (GSWP3) data set,^38^ span the same temporal range as previous studies (1995–2014) with a 6 h time resolution. Climate forcing data set parameters include precipitation (mm H_2_O/sec), incoming solar radiation (W/m^2^), temperature (K), pressure (Pa), winds (m/s), humidity (kg/kg), and downward longwave radiation (W/m^2^). The data sets of the land use and land cover follow the data sets of Land Use. Harmonization 2 (LUH2, luh.umd.edu/data.shtml) at present day,^39^ and other surface data sets follow the setting of CLM5.^40^ By incorporating a range of land unit data sets, our simulations capture the impacts of land use and land cover changes, including deforestation, agriculture, and urbanization. The specific simulations conducted in this study are listed in Table 1.

### 2.2 Inventory of Anthropogenic Hg Release for Rivers

Existing global inventories report total Hg releases without specifying the fractions partitioned between land and water,^15^ leaving source-specific aquatic release fractions highly uncertain. For example, the Kocman inventory^14^ estimates a lower-bound anthropogenic Hg release of ∼1000 Mg/yr to global rivers; however, directly applying this value to drive MOSART-Hg-WM in preliminary simulations yields unrealistically low Hg export to the oceans, indicating a mismatch between inventory assumptions and observed riverine fluxes. To address this limitation, we extend previous global riverine Hg assessments focused on estuaries and coastal fluxes (e.g., Liu et al.^1^ and Amos et al.^19^) to explicitly represent inland river channels and reservoirs/dams. Model performance is evaluated against riverine Hg observations reported in the literature. Building on this framework, we construct a global, grid-resolved inventory of Hg releases to surface waters by adopting the ASGM fraction of total anthropogenic Hg releases as defined in Kocman et al.^14^ Unlike prior source-based approaches,^14,41^ our inventory is constrained from an observational perspective, enabling a more realistic representation of Hg inputs to rivers, particularly in the absence of comprehensive statistics and reports from these sources.^15^

To constrain sector-specific release fractions and reduce these uncertainties, we developed an inverse estimation framework. First, to define the spatial distribution of aquatic Hg releases, we used atmospheric Hg emission inventories [e.g., Arctic Monitoring and Assessment Programme (AMAP) inventory,^42^ and Streets inventory^43^] as spatial proxies for anthropogenic sources to rivers. This approach is justified by evidence that major anthropogenic Hg emissions to the atmosphere, land, and water broadly share similar spatial patterns.^15^ To minimize inconsistencies where atmospheric and aquatic release pathways diverge, we limited the analysis to three core AMAP sectors, including industrial activities (INDS), energy production (POWERGEN), and artisanal and small-scale gold mining (ASGM). Sectors with weak or poorly aligned links to aquatic releases, such as the intentional use waste (INTWASTE) category in the AMAP inventory, were excluded.

Second, to quantify the specific release coefficients for these selected sectors, we applied a top-down constraint based on the global amount of Hg exported to oceans, approximately 1000 Mg/yr as derived from the river mouth monitoring data.^1^ The Hg releases from human-induced sources to rivers are categorized as direct and indirect releases (i.e., the short-term legacy Hg release). Direct release involves Hg discharged into rivers via such as urban sewage pipe networks, while indirect release entails Hg initially deposited in soil, mobilized by erosional processes, and transported to rivers over a period of one year. The sediment yield serves as a crucial parameter for determining indirect release. In this study, the sediment yield data set represents the mean sediment yield in 2010, estimated under the ***No Release Scenario*** (Table 1). The simulation of the sediment yield is decoupled from Hg cycling, so the sediment yield remains the same across all scenarios.

Given validated runoff and sediment simulations as boundary conditions, we iteratively calibrated the sector-specific release coefficients, i.e., the *Coef* terms in eqs 2–7, to match this global ocean Hg export target. Within this constrained source-sink framework,^25^ the derived coefficients for ASGM of direct releases are strictly higher than those for industrial sources, reflecting its direct aquatic discharge pathways, while INDS and POWERGEN share identical coefficients under the same simulation scenarios (Table 2). The grid-based anthropogenic Hg releases to rivers are calculated by the following equations

2 $${\mathrm{EI}}_{\mathrm{ASGM}}^{\mathrm{THg}}={\mathrm{AMAP}}_{\mathrm{ASGM}}^{\mathrm{THg}}\times {\mathrm{sed}}_{\mathrm{yld}}\times {\mathrm{coef}}_{\mathrm{ASGM}}^{I}$$

3 $${\mathrm{EI}}_{\mathrm{INDS}}^{\mathrm{THg}}={\mathrm{AMAP}}_{\mathrm{INDS}}^{\mathrm{THg}}\times {\mathrm{sed}}_{\mathrm{yld}}\times {\mathrm{coef}}_{\mathrm{INDS}}^{I}$$

4 $${\mathrm{EI}}_{\mathrm{POWERGEN}}^{\mathrm{THg}}={\mathrm{AMAP}}_{\mathrm{POWERGEN}}^{\mathrm{THg}}\times {\mathrm{sed}}_{\mathrm{yld}}\times {\mathrm{coef}}_{\mathrm{POWERGEN}}^{I}$$

where EI_ASGM_^THg^, EI_INDS_^THg^, and EI_POWERGEN_^THg^ represent the indirect release by ASGM, industrial, or power-generation sectors, respectively (unit: g/km^2^/yr); AMAP_ASGM_^THg^, AMAP_INDS_^THg^, and AMAP_POWERGEN_^THg^ represent the spatial distribution of release from different sectors (unit: g/km^2^/yr); sed_yld_ represents the sediment yield (unit: kg/m^2^/s); and coef_ASGM_^I^, coef_INDS_^I^, and coef_POWERGEN_^I^ represent the coefficient of Hg release associated with erosion of different sectors (unit: m^2^s/kg). The settings of the Coefs are given in Table 2.

5 $${ED}_{ASGM}^{THg}={AMAP}_{ASGM}^{THg}\times {coef}_{ASGM}^{D}$$

6 $${ED}_{INDS}^{THg}={AMAP}_{INDS}^{THg}\times {coef}_{INDS}^{D}$$

7 $${ED}_{POWERGEN}^{THg}={AMAP}_{POWERGEN}^{THg}\times {coef}_{POWERGEN}^{ID}$$

where ED_ASGM_^THg^, ED_INDS_^THg^, and ED_POWERGEN_^THg^ represent the direct release for varied sectors (unit: g/km^2^/yr); AMAP_ASGM_^THg^, AMAP_INDS_^THg^, and AMAP_POWERGEN_^THg^ represent the spatial distribution of release (unit: g/km^2^/yr); and coef_ASGM_^D^, coef_INDS_^D^, and coef_POWERGEN_^D^ represent the coefficient of direct Hg release by sectors (dimensionless).

8 $$E_{\mathrm{total}}^{\mathrm{THg}}={{\mathrm{EI}}_{\mathrm{ASGM}}^{\mathrm{THg}}+{\mathrm{EI}}_{\mathrm{INDS}}^{\mathrm{THg}}{+\mathrm{EI}}_{\mathrm{POWERGEN}}^{\mathrm{THg}}+\mathrm{ED}}_{\mathrm{ASGM}}^{\mathrm{THg}}+{\mathrm{ED}}_{\mathrm{INDS}}^{\mathrm{THg}}{+\mathrm{ED}}_{\mathrm{POWERGEN}}^{\mathrm{THg}}$$

where *E*_total_^THg^ represents the total anthropogenic Hg release to rivers, which is eventually used to drive the MOSART-Hg-WM model. The coefficients in eqs 2–7 are adjustable to fit the requirements of various scenarios (Table 2).

**Table 2:** Scenarios of Industrial Releases and Artisanal and Small-Scale Gold Mining (ASGM) Releases to Rivers^a^

			indirect release [Mg/yr (coef.)]			indirect release [Mg/yr (coef.)]			
				industrial			industrial		
scenarios	anthropogenic Hg budget (Mg/yr)	changes (Mg/yr)	ASGM	INDS	POWERGEN	ASGM	INDS	POWERGEN	% from ASGM
No Release	0	= 0	0 (0)	0 (0)	0 (0)	0 (0)	0 (0)	0 (0)	0
Low Release	1100	= No Release +1100	147 (0.6 × 10^–6^)	72 (0.82 × 10^–7^)	5 (0.82 × 10^–7^)	378 (0.35)	460 (0.08)	36 (0.08)	48%
Moderate Release	1500	= Low Release +400	232 (0.95 × 10^–6^)	84 (0.95 × 10^–7^)	6 (0.95 × 10^–7^)	378 (0.35)	748 (0.165)	59 (0.165)	41%
Revised AMAP Release	∼1500	≈ Moderate Release							
High Industrial Release	2000	= Moderate Release +500	232 (0.95 × 10^–6^)	343 (0.39 × 10^–6^)	25 (0.39 × 10^–6^)	378 (0.35)	949 (0.165)	74 (0.165)	30%
High ASGM Release	2000	= Moderate Release +500	367 (1.5 × 10^–6^)	132 (1.5 × 10^–7^)	9 (1.5 × 10^–7^)	475 (0.44)	949 (0.165)	74 (0.165)	42%

a The coef. refer to the coefficients in eqs 2 to 7, and the % of ASGM represents the percentage of anthropogenic Hg releases originating from ASGM.

Following a series of preliminary simulations, we find that a total anthropogenic Hg release of 1100–2000 Mg/yr provides the most realistic range for global riverine Hg simulations (Tables 2 and 3). This corresponds to an estimated global riverine Hg export to the oceans of 777–1275 Mg/yr, which is within ±25% of the previous estimate of ∼1000 Mg/yr reported by Liu et al.^1^ Our estimates assumed that ASGM accounts for 30–48% of total anthropogenic Hg inputs to rivers (Table 2). This range is consistent with the Kocman inventory assumption of ∼40%,^14^ and lower than the Global Mercury Assessment 2018 (GMA2018),^16^ which assumes ∼50% contribution from ASGM. In contrast, Horowitz et al.^41^ estimated a substantially higher ASGM contribution to water than those estimations in global scale, with approximately 80% of total Hg releases to water attributed to ASGM. Such high ASGM contributions would be particularly pronounced in regions with intensive ASGM activity, such as the Amazon, where simulations show strong sensitivity to the assumed ASGM emissions (refer to Section 3.5). Model sensitivity tests further show that relatively small shifts in ASGM fraction can induce disproportionate changes in riverine Hg export; for example, a 12% increase in ASGM contribution is related to a 32% increase in Hg fluxes in the Amazon (refer to Section 3.5).

**Table 3:** Fates of Riverine Mercury under Different Scenarios

scenarios	anthropogenic Hg releases (Mg/yr)	inputs of riverine Hg (Mg/yr)	outputs of riverine Hg (Mg/yr)	increased release^a^ (Mg/yr)	increased outputs (Mg/yr)	increased export/increased releases (%)	dam trapping rate (%)
No	0	418	248	0	–	–	41%
Low	1100	1498	777	1100	529	48%	48%
Moderate	1500	2005	1007	400	230	58%	50%
High Industrial	2000	2532	1146	500	139	28%	55%
High ASGM	2000	2436	1275	500	268	54%	48%
Baseline	∼1500	2032	1014	–	–	–	50%

a Increased release as compared with the previous one, such as “moderate–low = 400”, “high–moderate = 500”.

In this study, anthropogenic Hg releases to rivers have been modeled in several scenarios to encompass different release situations for the present day (Table 2). We consider five scenarios to analyze the uncertainties of anthropogenic Hg releases from industrial and ASGM sources to the rivers at present day (*Inventory Experiment*): (1) *Low Release* (1100 Mg/yr), (2) *Moderate Release* (1500 Mg/yr) (similar level with *Baseline Scenario*), (3) *High Industrial Release* (2000 Mg/yr, ASGM contribute 600 Mg/yr), (4) *High ASGM Release* (2000 Mg/yr, ASGM contribute 850 Mg/yr), and (5) *No Release* (0 Mg/yr). Other parameters in the model simulations, such as the number of dams, remain unchanged to ensure that the human-induced Hg release is the sole variable in the control experiments.

The *Low Release scenario* represents the unique period during the COVID-19 pandemic when industrial and ASGM releases are significantly reduced due to lockdown measures.^44,45^ The around −40% reduction in indirect ASGM releases relative to *Moderate Release* scenario reflects decreased human activities, such as agricultural activity during lockdown periods^46^ (Table 2). In contrast, direct ASGM emissions are assumed to remain unchanged across scenarios, as overall production is considered stable during lockdowns, with increased illegal mining activity offsetting partial disruptions to formal ASGM operations.^45^ The *Moderate Release scenario* represents a more realistic post-Minamata Convention condition with reduced anthropogenic Hg releases compared to the high-releases scenarios.^1^ However, it still retains relatively higher industrial (∼ +60%) and indirect ASGM releases (∼ +60%) than lockdown-period levels, reflecting the recovery of human activities following the pandemic.

The *High Industrial Release scenario* represents conditions around the year 2000, characterized by elevated industrial Hg releases (∼55% higher than the Moderate Release scenario), reflecting less effective environmental management and weaker regulatory controls.^19^ The *High ASGM Release scenario* represents an alternative pathway for the same period, in which industrial releases are moderately increased (∼30%), while ASGM activity is further enhanced, leading to a larger overall ASGM contribution (∼40%). The *High Industrial Release* and *High ASGM Release scenarios* share the same total global Hg release but differ in the relative contributions from industrial sources and ASGM. This design isolates the effects of sectoral composition on riverine Hg dynamics under a constant total Hg loading (refer to Section 3.5). The *No Release scenario* is an idealized case in which only erosional Hg inputs are considered under present-day conditions. Together, these scenarios allow assessment of the sensitivity of riverine Hg dynamics to variations in sector-specific anthropogenic inputs.

To better reproduce observed riverine Hg exports, we adopt the AMAP spatial distribution under the *Moderate Release scenario*, except in North America where the Streets inventory is applied (also under the *Moderate Release scenario*), as the AMAP pattern leads to overestimated Hg export in this region. This hybrid configuration is hereafter referred to as the *Revised AMAP Release scenario* and is used as the Hg input data set for the ***Baseline scenario*** in this study. A comparison of the uncertainty associated with these alternative spatial distributions is provided in Figure S3. The primary experiment (***Baseline scenario***) therefore employs total Hg release inputs of ∼1500 Mg/yr with this combined AMAP-Streets spatial pattern (Tables 1 and 2). This experiment facilitates a realistic representation of the present-day scenario by closely aligning with observational records.

### 2.3 Experimental Design

In this study, due to the absence of key parameters such as carbon concentration and plankton distribution in the MOSART model, we assume that Hg in rivers primarily exists in the particulate phase, partially following sedimentation and sediment resuspension processes to simplify the model (see Text S1). Despite these assumptions, accurately representing Hg fluxes from land to the ocean remains our primary goal. A series of grouped experiments are devised to evaluate sensitivities and uncertainties stemming from different processes: *Reservoir* (with or without reservoir), *Inventory* (spatial distribution following AMAP or Streets Atmosphere inventories), *Soil Hg* (soil Hg concentrations following Wang or Liu data set), and *Deposition* (with atmospheric deposition or not) (Table 1). Specifically, as for the *Reservoir Experiment*, we conduct paired sensitivity experiments to evaluate the role of reservoirs and dams in Hg sequestration and downstream release. Those sensitivity experiments include comparisons of *No Human-Induced Hg Release with/without Reservoir* (*NHHR w/wt R*) and *Baseline Human-Induced Hg Releases with/without Reservoir* (*BHHR w/wt R*) (Table 1).

In the *Soil Hg* and *Deposition Experiments*, we conduct paired experiments to test the sensitivities of soil Hg data sets and atmospheric deposition, respectively (Table 1). To address the uncertainties stemming from soil Hg, we conduct the *Soil Hg Experiments* involving various soil Hg concentration data sets (referred to as the Liu data set by Liu et al.^37^ and Wang data set by Wang et al.^36^). The Wang data set’s simulation outputs closely match previous estimation^1^ (refer to Text S2 *Uncertainty analysis*), so we apply it as the primary data set for baseline simulations. We tested the effect of dynamic soil Hg concentrations driven by contemporary atmospheric deposition inputs and erosion-driven losses (refer to *Deposition Experiments*, Table 1).

The *Deposition Experiment* is designed to isolate the short-term response of soil Hg to contemporary atmospheric deposition. In the dynamic configuration (*Deposition*), soil Hg concentrations are allowed to evolve during the 10-year simulation, increasing through atmospheric deposition inputs and decreasing via erosion. In the *without deposition* configuration, soil Hg concentrations are held constant and do not respond to atmospheric inputs over the simulation period. Both configurations start from the same prescribed soil Hg fields, which incorporate the steady-state natural Hg cycle together with the cumulative legacy of anthropogenic emissions relative to the preindustrial baseline. Therefore, the long-term accumulated Hg in soils is identical in both experiments. Therefore, long-term accumulated Hg in soil is identical in both experiments. The difference between the two experiments thus reflects only the short-term soil Hg adjustment driven by present-day deposition during the 10-year simulation window.

## 3 Results and Discussion

### 3.1 The Global Riverine Hg Sources and Sinks

Our modeling effort provides a global pattern of riverine Hg, including its sources, transport, and fate under different release scenarios (Figure 1). Previous estimations by Streets et al.^15^ indicated that global anthropogenic Hg releases to land and water are in total at ∼7300 Mg/yr (mean) in 2010. In this study, our results of baseline simulation suggested that about 20% of this amount enters river systems, amounting to roughly 1500 Mg/yr (mean, different release scenarios ranging from 1100 to 2000 Mg/yr) (Table 3). This magnitude may be reflected in recent isotope-based source-tracking studies.^48^ Of these human-induced Hg releases to rivers, 30%–48% originate from ASGM under different release scenarios. The gross Hg inputs to rivers under the Baseline scenario consist of erosional and human-induced Hg releases, totaling approximately 1900 Mg/yr, with around 30% of this contributed by erosional soil Hg fluxes (∼420 Mg/yr). Long-term legacy Hg sources from floodplains are accounted for through either erosion or river sediment resuspension processes, depending on whether the floodplains are submerged. Hg contamination from releases to land, such as historical Hg inputs to soils, is represented by present-day soil Hg concentration data sets.^36,37^ We also tested the effect of dynamic soil Hg concentrations driven by Hg deposition (+) and soil erosion (−) (refer to *Deposition Experiment*, Table 1). The results show that incorporating short-term Hg deposition produces riverine Hg budgets to the ocean that are similar to those obtained under the assumption of constant soil Hg concentrations (differences <5%, Figure S5). However, using the assumption of constant soil Hg concentration significantly reduces computational resources when running high-resolution simulations with CESM2; hence, we use a constant soil Hg assumption in the major simulations.

**Figure 1:**
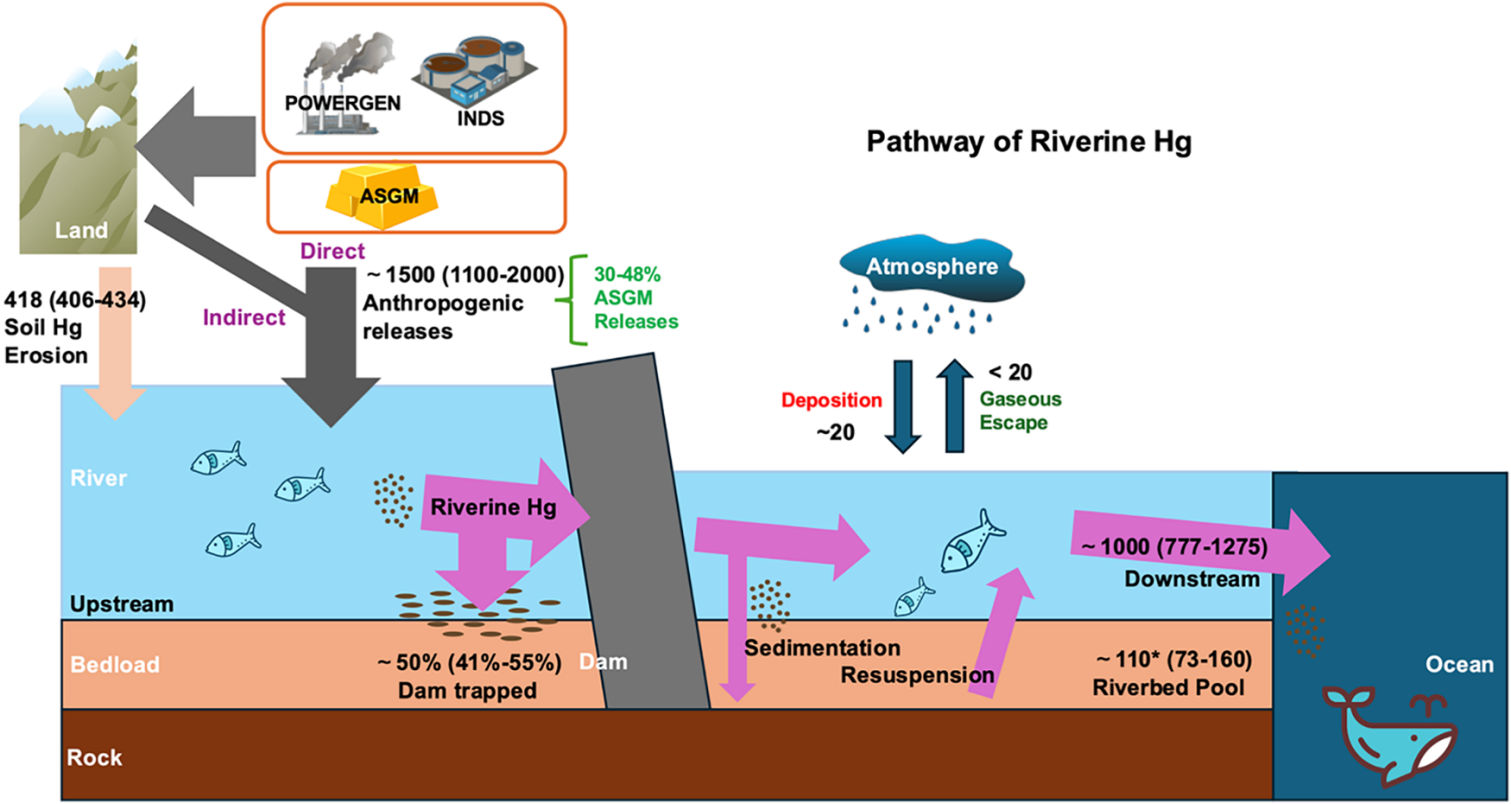
Pathways and fluxes of Hg in global rivers in 2010 under different release scenarios (unit: Mg/yr). Values in parentheses indicate the range around the mean. An asterisk (*) denotes riverbed Hg pools at the end of the 10-year simulation (excluding reservoirs), expressed in Mg. Anthropogenic Hg release categories include industrial activities (INDS), energy production (POWERGEN), and artisanal and small-scale gold mining (ASGM).

Atmospheric Hg deposition has limited short-term impacts on erosional Hg fluxes through changes in soil Hg concentration in the watershed, indirectly increasing erosional Hg, which aligns with the increasing soil Hg over time (refer to Text S2 Uncertainty analysis, *Deposition Experiment*). The long-term impacts of atmospheric Hg deposition on the watershed are accounted for in this simulation through the incorporation of soil Hg concentration changes from preindustrial to the present-day by using a data set of the present-day soil Hg concentration. Due to the barrier effect of dams and reservoirs, 41–55% of riverine Hg settles with sediments in these reservoirs (Table 3). Riverine Hg originating from ASGM sources has a higher likelihood of reaching the oceans, as reservoirs and dams are more strongly associated with industrial activity (e.g., regions with greater hydropower development and industrial infrastructure). As a result, the trapping efficiency is lower for ASGM-derived Hg, with a ∼7% reduction in trapping rate under the High ASGM Release scenario compared to the *High Industrial Release* scenario (Table 3).

In our simulations, contemporary atmospheric Hg deposition to the global land surface contributes an additional ∼20 Mg/yr of terrestrial Hg inputs to rivers, as represented in the Deposition Experiments. This contribution is negligible compared to other Hg sources (anthropogenic or erosional Hg) and primarily reflects a short-term legacy effect of recent atmospheric deposition. The established literature demonstrates that the global net air–water exchange over lotic systems is directed from the atmosphere into the water.^33,49^ Constrained by this net-depositional gradient and the magnitude of the atmospheric-derived terrestrial inputs, we deduce that gross gaseous Hg evasion from the river surface is minimal, bounded at less than 20 Mg/yr across the air–water interface. In-stream Hg sedimentation is computed dynamically from sediment fluxes and particulate Hg concentrations, explicitly accounting for resuspension during high-flow events. Over the short-term 10-year simulation period, approximately 110 Mg (mean) of Hg is deposited in riverbeds. Riverbed Hg constitutes a transient sink, easily mobilized by high-flow flood pulses. Notably, in-stream sedimentation remains substantially lower than reservoir trapping. This disparity is strictly hydrodynamic; free-flowing rivers maintain sufficient transport capacities to suspend particulate Hg, whereas reservoirs drastically reduce flow velocities, forcing highly efficient sediment and Hg settling. Ultimately, riverine Hg export to the oceans, defined as the net flux leaving each river network, reaches approximately 1000 (mean, range from 777 to 1275) Mg/yr.

### 3.2 Hg Inputs to the Global Rivers

The input of Hg to rivers is mostly contributed by soil erosion processes and human-induced releases, with the latter acting as the predominant source during the present day (Figures 1 and 2). Human-induced soil erosion, such as that resulting from deforestation, and long-term soil Hg accumulation, such as centuries of Hg deposition, are included in the soil Hg erosion process. In contrast, short-term contaminated soil erosion, for example, Hg deposited on land and subsequently mobilized into rivers within a single year, is treated as part of the human-induced Hg release process. We consider the *Baseline Scenario* (based on the *revised AMAP release* scenario) wherein the gross anthropogenic Hg release to rivers is 1500 Mg/yr, representing the present-day conditions (with ∼1000 Mg/yr, mean, global riverine Hg export fluxes from rivers to oceans by Liu et al.,^1^ Table 2). We categorize anthropogenic Hg releases into two categories to align with the AMAP inventory,^42,47^ including Industrial and ASGM (Figure 2). The spatial distributions of anthropogenic Hg sources in North America are based on the Streets inventory,^43^ with other continents on the AMAP inventory (refer to the Section 2). Hence, the higher industrial Hg releases are mostly located in East Asia, South Asia, North America, West Europe, and part of South Africa, which are aligned with atmospheric Hg emissions (Figure 2a). The ASGM Hg releases are mostly located in South and Central America, Southeast Asia, and South-Central Africa (Figure 2b).

**Figure 2:**
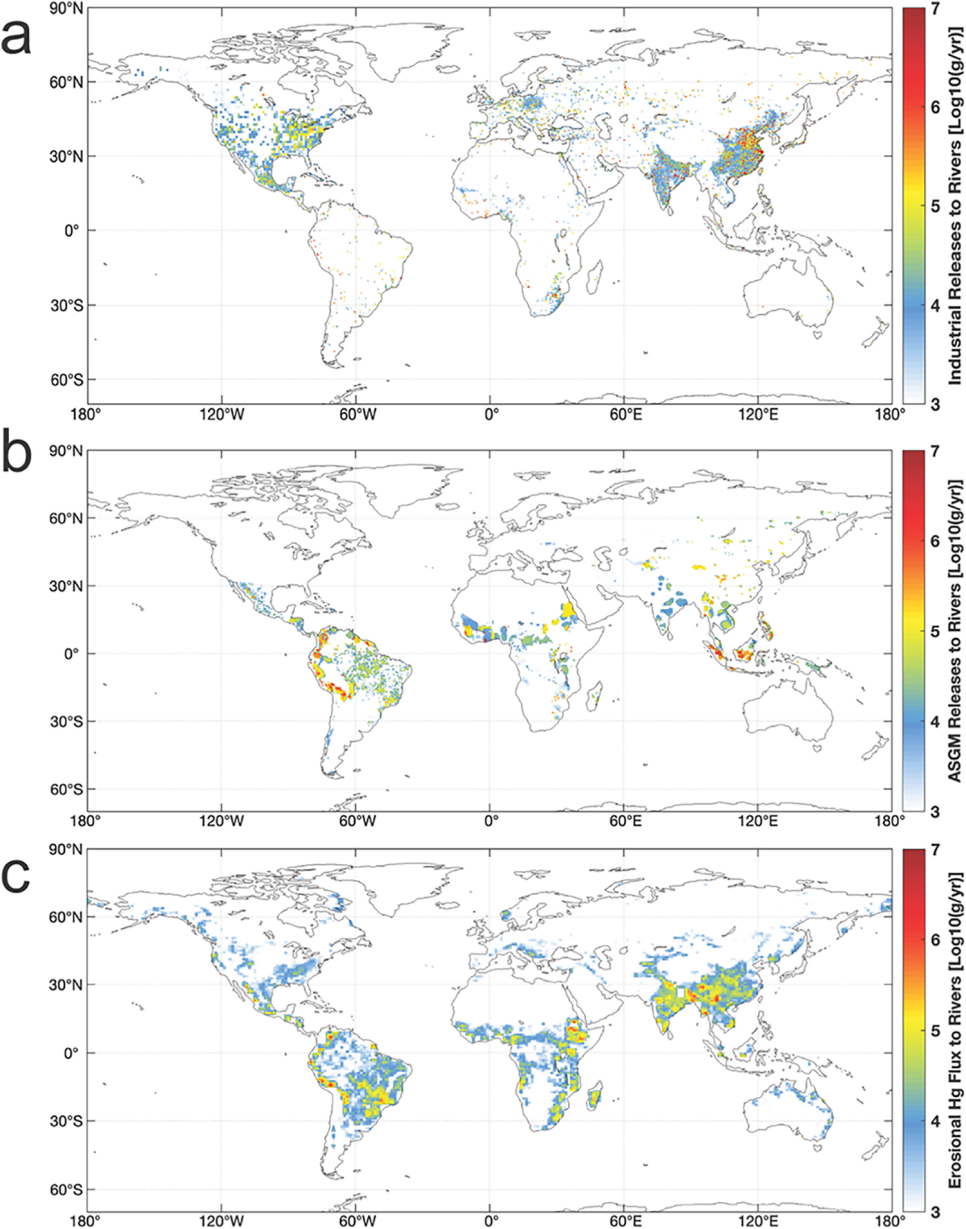
Input of Hg to global rivers. (a) Industrial releases to rivers, (b) artisanal and small-scale gold mining (ASGM) releases to rivers, (c) erosional Hg flux to rivers. Each data point represents a 0.5° × 0.5° latitude-longitude grid cell; therefore, Hg releases below 10^3^ g/yr may be reported as zero.

The contribution of soil Hg erosion to riverine Hg is relatively small (Figure 2c) and influenced by factors such as precipitation, vegetation, soil characteristics, slope, and runoff.^2,25,50^ In general, erosional Hg contributes 418 (406–434) Mg/yr to global rivers across the different release scenarios. This flux is independent of anthropogenic Hg releases and dam trapping processes, as erosion is parametrized as a separate natural pathway. Its spatial distribution and amounts are consistent with those simulated in our previous study for the preindustrial period, with ∼400 Mg/yr (mean).^25^ Comparing preindustrial and present-day simulations, we align land use, land cover, soil Hg, and land surface data sets with their respective periods to ensure consistent representation of erosional Hg fluxes across different time slices. The resulting difference in erosional Hg between the two periods is small (∼20 Mg/yr, mean), indicating limited sensitivity to elevated soil Hg stocks and climatic variations at the global scale. Regions adjacent to plateaus have higher erosional Hg fluxes, such as those near the Tibetan Plateau in East Asia and South Asia, as well as areas near mountains, such as those on the west coast of South America near the Andes Mountains. The model also simulates higher erosional Hg flux in the Amazon watershed during the present day (39.2 Mg/yr, mean) rather than in the preindustrial era (26.2 Mg/yr, mean) due to deforestation in the region.^25^ The change in erosional Hg flux from the preindustrial era to the present day is approximately 33% (mean) relative to present-day levels. This magnitude is consistent with the business-as-usual projection in Amazon by 2050 reported by Feinberg et al.,^51^ which also shows an increase of about 33% compared to present-day conditions. The simulated erosional Hg flux to rivers in Europe (including the UK) is approximately 1.3 Mg/yr (mean), which is lower than the previous estimate of 5.9 Mg/yr (mean) reported by Panagos et al.^52^ This discrepancy may stem from the coarse resolution of our simulation and the inclusion of mixed anthropogenic Hg contributions (such as agriculture) from earlier estimation.^52^

The contribution of soil Hg erosion to riverine Hg is relatively small (Figure 2c) and influenced by factors such as precipitation, vegetation, soil characteristics, slope, and runoff.^2,25,50^ In general, erosional Hg contributes 418 (406–434) Mg/yr to global rivers across the different release scenarios. This flux is independent of anthropogenic Hg releases and dam trapping processes, as erosion is parametrized as a separate natural pathway. Its spatial distribution and amounts are consistent with those simulated in our previous study for the preindustrial period, with ∼400 Mg/yr (mean).^25^ Comparing preindustrial and present-day simulations, we align land use, land cover, soil Hg, and land surface data sets with their respective periods to ensure consistent representation of erosional Hg fluxes across different time slices. The resulting difference in erosional Hg between the two periods is small (∼20 Mg/yr, mean), indicating limited sensitivity to elevated soil Hg stocks and climatic variations at the global scale. Regions adjacent to plateaus have higher erosional Hg fluxes, such as those near the Tibetan Plateau in East Asia and South Asia, as well as areas near mountains, such as those on the west coast of South America near the Andes Mountains. The model also simulates higher Hg erosion flux in the Amazon watershed during the present day (39.2 Mg/yr, mean) rather than in the preindustrial era (26.2 Mg/yr, mean) due to deforestation in the region.^25^ The change in erosional Hg flux from the preindustrial era to the present day is approximately 33% (mean) relative to present-day levels. This magnitude is consistent with the business-as-usual projection in Amazon by 2050 reported by Feinberg et al.,^51^ which also shows an increase of about 33% compared to present-day conditions. The simulated erosional Hg flux to rivers in Europe (including the UK) is approximately 1.3 Mg/yr (mean), which is lower than the previous estimate of 5.9 Mg/yr (mean) reported by Panagos et al.^52^ This discrepancy may stem from the coarse resolution of our simulation and the inclusion of mixed anthropogenic Hg contributions (such as agriculture) from earlier estimation.^52^

### 3.3 Impacts from Reservoirs/Dams to Riverine Hg

Our findings suggest that reservoirs/dams can largely influence the global gross riverine Hg budget by reducing transport flux by 40–50% (Figures 1 and 3). These Hg trapping rates are consistent with previous estimates of global sediment retention (∼50%, mean).^53,54^ This agreement supports the robustness of the modeled Hg trapping estimates, given the similar transport behavior of Hg and sediments, particularly the dominance of particulate-bound Hg in rivers. The Hg form may change after settling into the bed of reservoirs, because of methylation and/or change to the dissolved phase, but in this study, we assume the Hg fluxes are only impacted by resuspension at this stage due to the relatively smaller fluxes related to the biological and other activities.^55^ Reservoirs and dams play roles in reducing riverine Hg transport fluxes and SPM in the downstream portions of rivers.^53,54^ These are important components of spatial variabilities for riverine Hg transport fluxes along channels. The increasing trend of Hg flux from upstream to downstream can be interrupted by reservoirs and dams, which trap Hg from upstream input and sharply decrease the flux. The Hg flux increases from the decreased levels downstream of a dam to higher levels before reaching the next dam.

**Figure 3:**
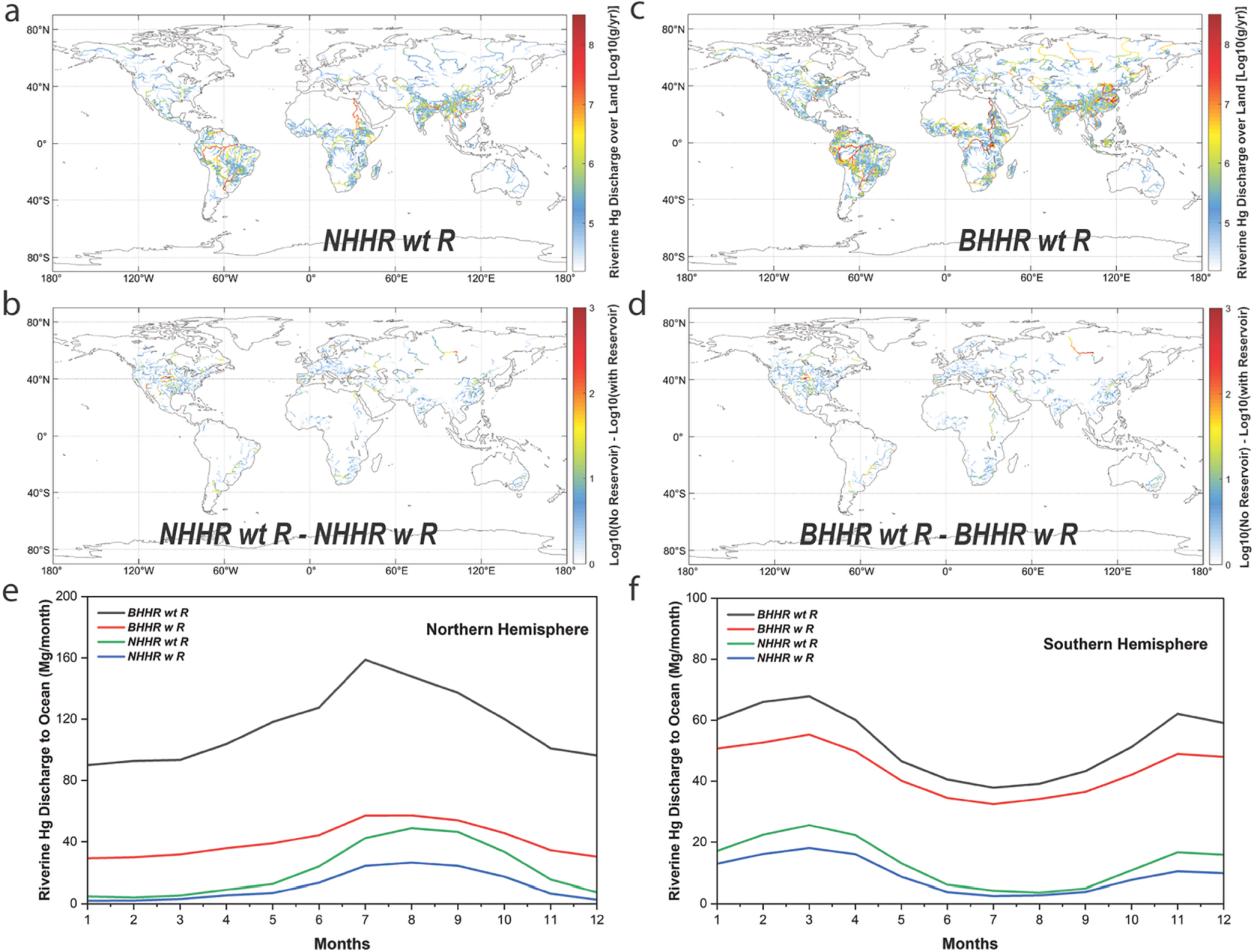
Impact of reservoirs and dams on riverine Hg transport. (a) Riverine Hg transport fluxes in global rivers under *No Human-induced Hg Release without Reservoir scenario (NHHR wt R)*. (b) Differences of riverine Hg transport fluxes between *No Human-induced Hg Release with/without Reservoir (NHHR w/wt R)*. (c) Riverine Hg transport fluxes in global rivers under *Baseline Human-induced Hg Releases without Reservoir scenario (BHHR wt R)*. (d) Differences of riverine Hg transport fluxes between *Baseline Human-induced Hg Releases with/without Reservoir (BHHR w/wt R)*. (e) Monthly riverine Hg export fluxes to oceans in Northern Hemisphere. (f) Monthly riverine Hg export fluxes to oceans in Southern Hemisphere. Each data point represents a 0.5° × 0.5° latitude-longitude grid cell.

In the presence of reservoirs, total riverine Hg export fluxes to the oceans decrease by 41% (mean) under scenarios without anthropogenic Hg releases, declining from 417 Mg/yr (*NHHR wt R*) to 248 Mg/yr (*NHHR w R*). If we consider both the erosional Hg inputs and anthropogenic Hg releases, reservoirs/dams reduce Hg fluxes to oceans by 50% from 2021 Mg/yr (*BHHR wt R*) to 1014 Mg/yr (*BHHR w R*). The difference between the two pairs of runs reflects the large regional variabilities associated with the impact of reservoirs (Figure 3b,d). When examining specific case studies of anthropogenic Hg releases (*BHHR w/wt R*), the riverine Hg exports of Yangtze, Yellow, Parana, and Niger rivers have been reduced by 77%, 75%, 63%, and 61%, respectively. This variability in Hg reduction at the local level is a reflection of the number of reservoirs or/and the portion of mega reservoirs such as the Three Gorges Dam and Aswan High Dam. Indeed, some rivers show limited impacts from water management if there are fewer reservoirs and/or fewer mega reservoirs along the river. For example, the Congo River, Amazon River, and Ganges-Brahmaputra Delta have experienced reductions in riverine Hg export fluxes of less than 1%, 1%, and 17%, respectively. As for the no-human-induced Hg release scenarios, the pattern is similar to the above regions (Figure 3b,d).

Reservoirs regulate river flooding and consequently the seasonal fluctuations of Hg fluxes, even though we assume that the human-induced Hg releases to rivers are constant during the various seasons in the simulations. For example, flood pulses driven by increased runoff and larger soil erosional fluxes during the wet season contrast with reduced fluxes during the dry season, thereby altering the soil Hg erosion fluxes. The differences between monthly global Hg exports have been reduced from 22.0 to 52.5 Mg/month (minimum to maximum, *NHHR wt R*) to 12.5–29.3 Mg/month (*NHHR w R*), with the amplitude reduced by 45% from 30.6 to 16.8 Mg/month. The monthly Hg export also decreased from 150.3 to 196.6 to 74.6–94.3 Mg/month under the *BHHR w/wt R*, with amplitude reduced by 58% from 46.3 to 19.7 Mg/month. The trapping effects of dams are more pronounced in the Northern Hemisphere (Figure 3e,f), attributed to the larger landmass and number of rivers and reservoirs as compared to the Southern Hemisphere.^35,56^ Furthermore, this effect is amplified by the larger anthropogenic Hg releases, leading to obvious differences in riverine Hg export fluxes during the summer in the Northern Hemisphere (Figure 3e). In conclusion, the reservoirs/dams reduce the effects of seasonal changes of riverine Hg.

### 3.4 River Hg Transport Fluxes and Export Fluxes

The modeled global riverine Hg export flux to oceans/lakes is ∼1000 Mg/yr (mean) in the *Baseline scenario (also the BHHR w R)* (Figures 1 and 2). This export demonstrates pronounced spatial variability, with significantly higher fluxes discharged along the coasts of Asia and South America compared to other global regions. The top ten largest riverine Hg exporters contribute 51% (506 Mg/yr, mean) of the global discharge, with the highest rivers including the Amazon (202 Mg/yr, mean), followed by the Congo (87 Mg/yr, mean), Yangtze (52 Mg/yr, mean), and Ganges-Brahmaputra Delta (51 Mg/yr, mean) (Figure 4). These large rivers dwarf the export fluxes from smaller rivers in these regions (less than 30 Mg/yr), especially along the north coast of South America, the Indian Peninsula, eastern China, and Southeast Asia. The denser river networks in East Asia, South America, and Central Africa could result in higher export Hg fluxes to oceans due to the joining of the rivers within the large watershed. For example, the Ganges River and the Brahmaputra River share the same estuary.

**Figure 4:**
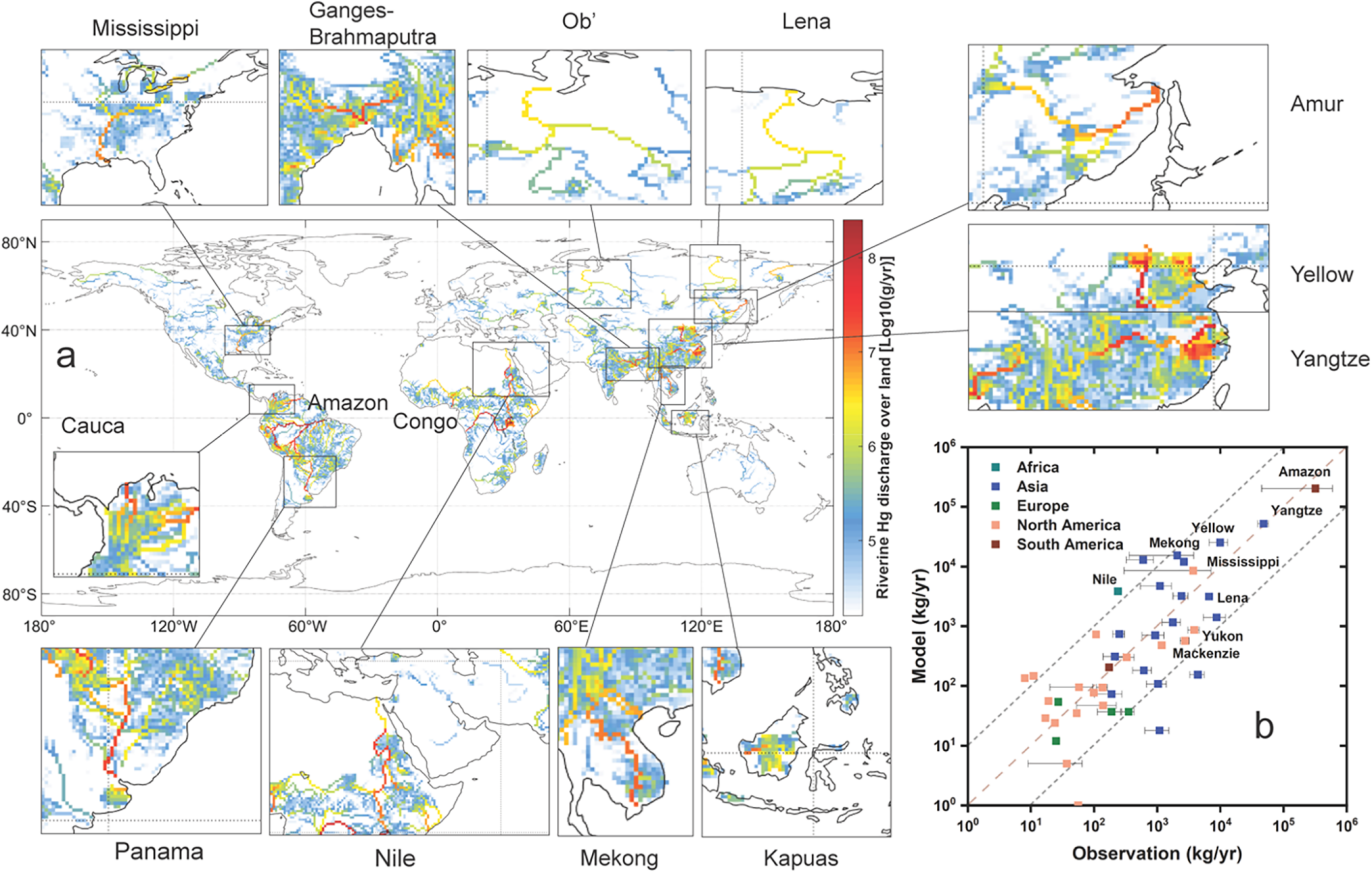
Riverine transport and export Hg fluxes in global rivers. (a) Spatial patterns of riverine Hg transport fluxes with zoomed regions of major rivers; (b) model results of riverine Hg export fluxes versus observation records on continents. Each data point represents a 0.5° × 0.5° latitude-longitude grid cell.

The modeled riverine Hg transport fluxes exhibit large spatial variations and seasonal variability (Figures 4 and S1). Generally, the Hg transport flux in many watersheds increases downstream along the main channel as tributaries merge, reaching their maximum at the river mouths, if there are no major dams. This pattern reflects the continuous input of Hg from wastewater and soil erosion in their watersheds and the high transport efficiency of SPM by rivers.^25^ For example, riverine transport Hg fluxes increase in the upstream stretches of the Three Gorges Dam (0–24 Mg/yr), and this trend resumes downstream of the Three Gorges Dam in the channels of the Yangtze River (4–52 Mg/yr) (Figure 4). The Panama and Mekong rivers also have increased riverine transport Hg fluxes along the channels and reach a maximum flux at the river mouth, but this trend is less influenced by dams.

Rivers with higher anthropogenic releases and/or erosion in their watersheds can easily maintain higher Hg transport fluxes in their channels, such as the Amazon, Congo, Yangtze Rivers, and Ganges-Brahmaputra Delta (Figures 2 and 4). For example, the highest riverine transport Hg flux in the Amazon River (mean = 201.7 Mg/yr near its mouth) is caused by the effect of ASGM releases and erosional Hg flux to the watershed (152.5 and 32.2 Mg/yr, mean, respectively). However, industrial Hg releases in the Amazon watershed are below 10 Mg/yr. As a result, the total Hg inputs to the Amazon watershed (152.5 + 32.2 + 10 = 194.7 Mg/yr, and nearly 50% trapping by reservoirs) are lower than the simulated riverine Hg export to the ocean (201.7 Mg/yr, mean). This imbalance suggests additional contributions from internal remobilization (e.g., resuspension of riverbed sediments) and/or external Hg inputs from upstream or adjacent regions that are not fully accounted for within the watershed budget. Similarly, ASGM Hg releases contribute substantially to both riverine transport and export fluxes in the Congo River. In contrast, higher riverine export Hg fluxes of the Yangtze River (52 Mg/yr at the mouth, mean) and Ganges-Brahmaputra Delta (51.2 Mg/yr at the mouth, mean) are caused by the higher industrial releases (100.7 and 37.8 Mg/yr, mean, respectively) and erosional Hg flux (from Himalayas to the ocean; 23.6 and 49.6 Mg/yr, mean, respectively) in these regions. Indeed, the watershed of the Yangtze River includes the most industrialized regions of eastern China (Figure 2a). Interestingly, some smaller rivers with relatively limited watershed areas, such as the Kapuas River (14.6 Mg/yr at the mouth, mean), exhibit comparatively high riverine Hg export fluxes due to intense ASGM activity (Figures 2b and 4), consistent with findings from local studies.^57^

Riverine export Hg fluxes estimated by the model agree well with the observation of riverine Hg export fluxes in river mouths (Figure 4b). The observational data set includes 44 rivers from various continents spanning 5 orders of magnitude with a mean flux of 1 × 10^3.99±3.9^ kg/yr. The model (mean flux is 1 × 10^3.90±3.7^ kg/yr) represents the observations with a high coefficient of determination (*R* ^2^ = 0.97, *p* < 0.01, Figure 4b). The model results of riverine Hg export fluxes agree well with observation records for many major rivers, such as the Yangtze (model vs observation: 52 vs 48 Mg/yr, mean), Ob′ (3.2 vs 2.4 Mg/yr, mean). Although the model has performed well in simulating erosion of the small rivers,^30^ because of coarse anthropogenic releases, the model has a higher bias for smaller rivers, such as the Kolyma (model vs observation: 4.7 vs 1.1 Mg/yr, mean), Mackenzie (2.6 vs 0.6 Mg/yr, mean) and Yukon Rivers (3.8 vs 0.9 Mg/yr, mean). These rivers exhibit relatively lower riverine export Hg fluxes compared to larger rivers but are more sensitive to anthropogenic Hg releases within their watersheds. Even small increases in anthropogenic Hg input can substantially elevate the Hg concentrations and fluxes of smaller rivers. For instance, discrepancies in releases in large watersheds may be mitigated by the average effect among grids (and/or tributaries) within the same watershed, but such an effect is less likely to occur in smaller watersheds. Furthermore, the model agrees with observed riverine Hg transport fluxes along river channels, although such observations are available for only a limited number of major rivers. For example, the model (24 Mg/yr, mean) closely resembles the observation (26 Mg/yr, mean) from the upstream portions of the Three Gorges Dam, as well as reaches immediately downstream (4.7 and 4.4 Mg/yr, mean).^20^

### 3.5 Impacts of Human-Induced Hg Releases

By comparing riverine Hg input and export fluxes across different release scenarios, we assess how rivers respond to variations in both the magnitude and sectoral composition of anthropogenic Hg releases. The results show that higher anthropogenic Hg releases lead to increased riverine Hg transport and export to the oceans (Figure 5e–g). Higher anthropogenic Hg releases increase Hg inputs to rivers, whereas the contribution from soil erosion remains stable across scenarios, because initial soil Hg conditions and climatic forcing are identical during the simulations. Note that the release scenarios isolate only annual anthropogenic Hg inputs to rivers; their long-term effects on soil pools and broader earth system components are not represented in the short-term simulations conducted in this study. The fate of Hg released from anthropogenic sectors, including riverine inputs and export to the oceans, varies across scenarios. For instance, despite comparable levels of total releases between *High Industrial Release* and *High ASGM Release Scenarios*, the former exhibits a lower export fraction (Figure 5f). With the *Moderate Release scenario* as a reference, only 28% of the increased industrial releases are ultimately transported to oceans for the *High Industrial Release* scenario (Table 3), but 54% are exported for the *High ASGM Release scenario*.

**Figure 5:**
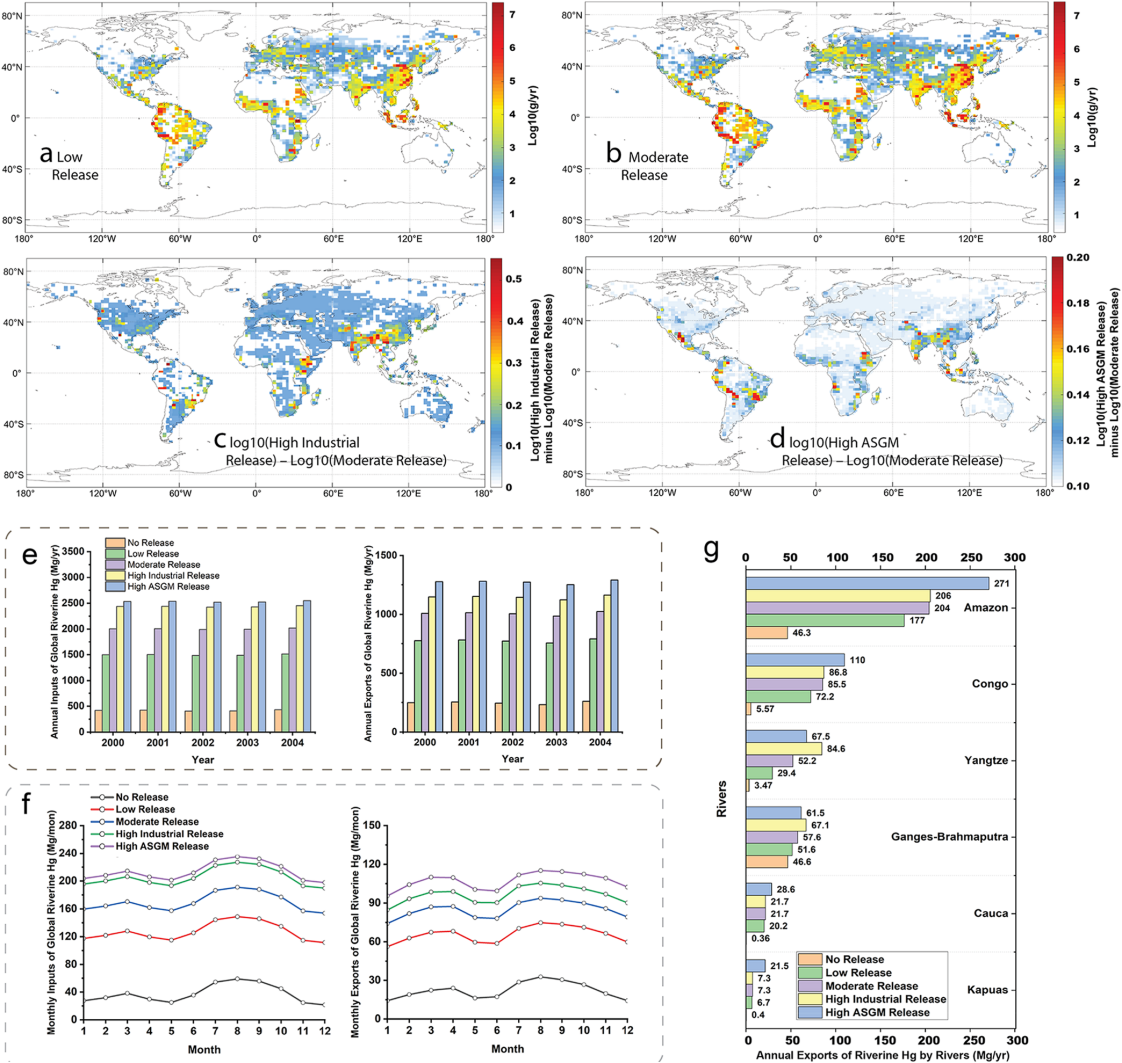
Impacts of anthropogenic Hg releases on riverine Hg fluxes under different release scenarios. (a) Anthropogenic Hg releases under the Low Release scenario; (b) anthropogenic Hg releases under the Moderate Release scenario; (c) differences in anthropogenic Hg releases between the High Industrial Release and Moderate Release scenarios (log10 scale); (d) differences in anthropogenic Hg releases between the High artisanal and small-scale gold mining (ASGM) Release and Moderate Release scenarios (log10 scale); (e) annual Hg inputs and outputs in global rivers; (f) monthly Hg inputs and outputs in global rivers; and (g) annual exports of riverine Hg to the oceans for selected rivers.

Higher industrial releases contribute to higher riverine Hg export fluxes in the rivers of East Asia and Southeast Asia, while increased ASGM releases contribute to the higher riverine Hg export fluxes in the Amazon River watershed, Central Africa, and Southeast Asia (Figure 5). These differences in riverine Hg transport and export fluxes are attributed to the differing water management strategies in various regions. For instance, the Amazon River has relatively more active ASGM activities and fewer reservoirs/dams and/or fewer mega reservoirs/dams in its watershed, causing higher export fractions.^58^ In contrast, the Yangtze River has higher industrial releases in its watershed and impacts from reservoirs and dams (Figure 3B). Indeed, regions with many industries often implement more extensive water management practices to provide water and hydroelectricity for reservoirs.^59^ This shift in water management could result in lower riverine Hg export fractions of industrial releases in those regions (Figure 5G).

### 3.6 Sink of Hg in Reservoirs/Dams

Increased Hg releases from human activities lead to higher Hg accumulation rates in reservoirs, which is the major sink for riverine Hg (Figures 1 and 6). Globally, Hg accumulation rates in reservoirs rise approximately 6-fold from 14 Mg/month (mean) in the *No Release scenario* (representing preindustrial era) to 84 Mg/month (mean) in the *Baseline Release scenario* (representing present-day). The contribution of different sectors (e.g., industrial vs ASGM sources) to Hg releases has a smaller effect on accumulation rates than riverine Hg transport and export fluxes, as shown by similar rates in the *High Industrial Release* (106 Mg/month, mean) and *High ASGM Release* (104 Mg/month, mean) scenarios. Because human-induced Hg releases are assumed to remain constant throughout the year and dominate over natural sources, the overall increase in Hg accumulation exhibits limited seasonal variation (Figure 5f). Differences among release scenarios on seasonal time scales are instead controlled mainly by variability in erosional Hg fluxes. As a result, anthropogenic inputs generate relatively stable monthly fluxes (Figure 6A). Reservoir storage capacities are designed for water and/or sediments during construction; therefore, the Hg storage capacity is not explicitly defined and remains uncertain. The storage estimates shown in Figure 6 are thus based on an idealized representation of reservoir behavior while the numbers of reservoirs are assumed to be constant during simulations.

**Figure 6:**
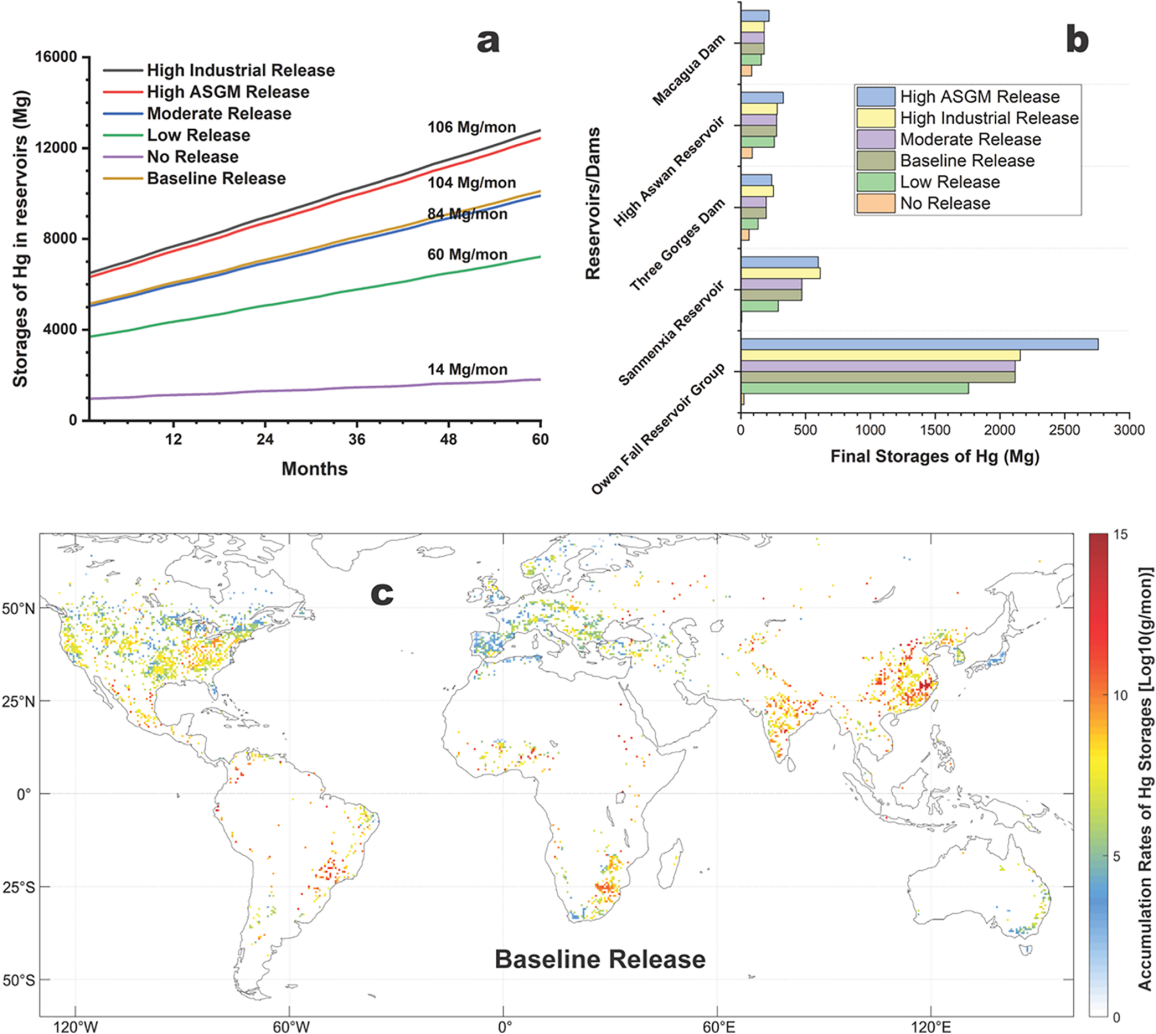
Reservoir storage of riverine Hg. (a) Monthly accumulation of Hg in global reservoirs; the starting point is defined by a five-year reservoir spin-up period, with reservoir Hg storage initialized to zero at the beginning of the simulation. (b) Final Hg storage in large reservoirs and dams over the 10-year simulation period. (c) Accumulation rates of Hg storage in global reservoirs and dams under the *Baseline Release scenario*. Note that the final Hg storage shown in panel (b) represents reservoir Hg storage at the end of the simulation period only. ASGM = Artisanal small-scale gold mining.

Reservoirs exhibit obvious regional variability in the rates of Hg accumulation, which is influenced by reservoir density and watershed Hg releases (Figure 6c). In East and South Asia, higher reservoir densities combined with substantial industrial Hg releases (Figure 2a) result in elevated Hg accumulation rates. In contrast, the United States, despite its high reservoir density, has relatively lower Hg releases (Figure 2a,b), leading to low to moderate accumulation rates. Reservoirs in parts of Southern Africa and South America exhibit high accumulation rates, consistent with elevated Hg releases from ASGM activities (Figures 2b and 6c). Meanwhile, southern Europe, with a high density of reservoirs but lower regional Hg releases, shows relatively lower Hg accumulation rates.

Several specific reservoirs illustrate the dominance of human-induced Hg releases on accumulation patterns. For example, the Owen Falls Reservoir Group (upstream of the Nile), the High Aswan Dam (Nile), the Sanmenxia Reservoir (Yellow River), the Three Gorges Dam (Yangtze River), and the Macagua Dam (Caroní River) show substantial increases in Hg accumulation between *No Release* to *Low Release* scenarios, with even larger increases from *Low* to *Moderate* or *High Release* levels (Figure 6b). Note that reservoir Hg storage is initialized to zero at the start of the 10-year simulations, and the reported values represent the accumulated storage at the end of the simulation period for each release scenario. Sectoral contributions also play a critical role: while reservoirs like the Sanmenxia and the Three Gorges experience greater accumulation under the *High Industrial Release* scenario, those such as the Owen Falls Reservoir Group see much higher accumulation rates under the *High ASGM Release* scenario than the *High Industrial Release* one.

The global scarcity of continuous in-channel observations and explicit reservoir Hg flux monitoring severely limits direct large-scale model validation. For instance, while recent studies have successfully identified reservoir trapping effects by comparing Hg fluxes across different time periods,^60^ the absence of concurrent in-channel data prevents the precise quantification of reservoir trapping ratios. Furthermore, although detailed long-term monitoring has accurately constrained Hg pools in specific reservoirs,^61^ these localized systems are often too small in volume and catchment area to serve as viable comparison points for a global Earth System Model operating at ∼1° spatial resolution.

Given these empirical limitations and the extreme rarity of comprehensive mass-balance data sets, the trapping efficiency of the few extensively monitored mega reservoirs serves as our most viable validation proxy. For example, our simulation shows that activating the dam module reduces riverine Hg transport in the upper Yangtze River by 71% (mean). This value is consistent with field-based mass-balance estimates for the Three Gorges Dam, which indicate a persistently high trapping efficiency of around 70% (mean) across multiple monitoring years (e.g., 2016, 2018, and 2021).^2,20^ These years are not selected to highlight a specific peak condition; rather, they are representative observations spanning different hydrological conditions, all of which consistently show similarly high retention in the Yangtze watershed. The scale gap between localized biogeochemical processes and global modeling can be bridged through a concerted effort. For example, expanded global observational networks in river systems, with a critical emphasis on continuous in-channel monitoring, can help to better constrain future macroscale assessments.

## 4 Environmental Implications

Anthropogenic Hg releases dominate riverine Hg delivery from upstream to downstream and export into inland lakes and/or oceans.^11,62^ However, measuring riverine Hg fluxes is challenging due to rapid changes in runoff parameters and considerable variability in Hg concentrations worldwide. The lack of historical data also hinders the proper evaluation of the trends of riverine Hg export and the contribution of anthropogenic Hg releases, posing management challenges, particularly for international rivers such as the Mekong, Jordan, Indus, Niger, and Nile Rivers.^63,64^ Our estimation provides insights into understanding regional contributions of anthropogenic Hg releases to downstream rivers and oceans. The Hg concentrations in the rivers have a strong connection to the levels of fish Hg;^65,66^ hence, our simulations could help to understand the Hg pollution status in the rivers and coastal environment, which is vital for fishing and aquaculture. Due to the unique environment of estuaries, where freshwater mixes with saltwater, most particulate-phase Hg settles in this region before transport along the coastline under the influence of physical forces, including the Coriolis effect.^67^ Previous studies estimate Hg burial in global continental shelf regions at ∼1290 Mg/yr (mean),^68^ with riverine inputs representing a major contributing source to coastal Hg sedimentation.^1^ Higher riverine Hg fluxes under High anthropogenic Hg release scenarios would enhance Hg burial in coastal systems, potentially creating additional hotspots for Hg methylation and accumulation within coastal food webs.

## Supplementary Material



## Data Availability

All data needed to evaluate the conclusions in the paper are present in the paper and/or the Supporting Information. All code is available at http://ebmg.online/mercury/. The data sets for model running of this study are available from the corresponding author upon reasonable request or download from CESM2 websites and E3SM websites. The raw data that support the findings of this study are available from the corresponding author upon reasonable request.

## Notes

Calculation of uncertainties associated with different Hg forms in riverine processes; uncertainty analysis; implications for Hg cycling and sustainability science; seasonal fluxes of riverine Hg discharge over land; comparisons of estimated sediment loads with previous estimates; comparisons of modeled riverine Hg export fluxes with observations under different inventory experiments; comparisons of model results based on different soil Hg concentration data sets; comparisons of model results with and without dynamic soil Hg and atmospheric Hg deposition; and compiled data on Hg forms in global rivers and the proportion of particulate-phase Hg relative to total Hg (PDF)
