# Supplementary material for: Tracing Mercury from Land to River: Global Sources, Retention, and Implications for Sustainability
Source: Environ Sci Technol. 2026 Jul 25;60(31):21885–900. doi: 10.1021/acs.est.6c03367 (PMC13472080; doi:10.1021/acs.est.6c03367)
Supplement: Supplementary file 1 [file es6c03367_si_001.pdf]

Supporting Information for

Tracing mercury from land to river: global sources, retention, and implications for sustainability

Dong Peng[1,2], Zeli Tan[3], Peipei Wu[4], Ruirong Chang[2], Shaojian Huang[2], Peng Zhang[2], Yujuan Wang[2], Zhengcheng Song[2], Yanxu Zhang[5*], Ting Lei[6], Maodian Liu[7], Beth A Middleton[8], Jianhua Gao[1], Junguo Liu[9], Guangchun Lei[6], Shu Tao[7,10]

[1]School of Geography and Ocean Science, Ministry of Education Key Laboratory for Coast and Island Development, Nanjing University, Nanjing, 210023, China

[2]School of Atmospheric Sciences, Nanjing University, Nanjing, 210023, China

[3]Pacific Northwest National Laboratory, Richland, WA, 99354, USA

[4]Scripps Institution of Oceanography, University of California San Diego, La Jolla, CA, 92093 USA

[5]Department of Earth and Environmental Sciences, Tulane University, New Orleans, LA, 70118, USA

[6]School of Ecology and Nature Conservation, Beijing Forestry University, Beijing, 100083, China

[7]College of Urban and Environmental Sciences, Peking University, Beijing, 100871 China

[8]U.S. Geological Survey, Wetland and Aquatic Research Center, Lafayette, LA, 70506 USA

[9]Henan Provincial Key Laboratory of Hydrosphere and Watershed Water Security, North China University of Water Resources and Electric Power, Zhengzhou, 450045, China

[10]School of Environmental Science and Engineering, Southern University of Science and Technology, Shenzhen, 518055, China

*Corresponding author: Yanxu Zhang (yzhang127@tulane.edu)

The supporting information contains:

Supporting text S1 to S3
Figures S1 to S5
Tables S1
Supporting Information References
Pages S1 to S19

Supporting Text

Text S1 The calculation of uncertainties from the Hg forms in the river's process

The "proportion" of particulate-phase Hg in global rivers varies significantly due to limited mechanistic research and insufficient datasets, making it challenging to accurately represent these dynamics in global river models. Observational studies, such as the observations in Yukon River, Schuster, et al. [1] observed: "*The vast majority (90%) of Hg export is associated with particulates*" and in Mackenzie River, Leitch, et al. [2] observed: "*The majority of the total Hg flux is carried in the form of particulate Hg, which accounts for 73–87% of the annual Hg flux in the past 3 yr*". Besides, in global estimations, Liu, et al. [3] recognized: "*Most riverine Hg is transported to the ocean associated with particles* [4]" and Amos, et al. [5] expressed a similar perspective, assuming that more than 95% of THg in rivers is particulate phase: "*Limited observations available suggest that rivers are highly contaminated ([Hg(D)] = 50−400 pM* [6] *and [THg] = [Hg(D)] + [Hg(P)] > 6000 pM* [7]*). We use [Hg(D)] = 50 pM as a conservative estimate.*"

Regions with dissolved phases as the majority of the total Hg (THg) are highly likely to be confined to local scales. For example, Kocman, et al. [8] reported that: "*The relatively high share of dissolved Hg forms at these sites is attributed to mercury leaching from ore residues and calcines, desorption from the particulate phase, and complexation with different organic and inorganic ligands present in water*." Indeed, a larger dissolved proportion than the particulate phase is typically observed in rivers with very low THg concentrations (Table S1). In rivers with higher THg concentrations, the particulate phase predominantly dominates. This observation is supported by earlier experimental studies, such as Lu, et al. [9], which reported: "*After spiking with mercury, the particulate mercury rose rapidly and reached over 70% of the total mercury (in the controlled experimental ecosystems).*"

Due to the absence of a biogeochemical module in the MOSART model within the CESM2 framework, Hg form changes and their impacts on riverine Hg dynamics are not accounted for in the simulations. In this study, we assumed that riverine Hg is entirely in the particulate phase, which introduces uncertainties to the model results. To address these uncertainties in both the riverine Hg budget exported to the ocean and the estimated inventory of human-induced Hg releases to rivers, we apply the below equations.

We conceptualize the global riverine Hg system as a box model, where riverine export follows mass balance principles. In this framework, the output of riverine Hg is divided into two components: losses and exports. Particulate Hg losses are primarily attributed to dam trapping, while dissolved Hg losses may result from processes such as water use and evaporation. Consequently, the following equation is applied:

$$\mathrm{PHg}_{\mathrm{flux}} \times \left(1 - Rate_{dam\ trapping}\right) + \mathrm{DHg}_{\mathrm{flux}} \times (1 - LossRate) = \mathrm{THg}_{\mathrm{export\ flux}} \quad \text{(e.S1)}$$

Where PHg_{flux} is the particulate phase Hg (PHg) flux in global rivers (Mg/yr), the $Rate_{dam\ trapping}$ is the global average dam trapping rate (%), DHg_{flux} is the dissolved phase Hg (DHg) flux in global rivers (Mg/yr), the $THg_{export\ flux}$ is the total Hg flux in the global rivers' export to the ocean (Mg/yr), and the LossRate is the loss rate of DHg_{flux} in global riverine Hg processes (%). The loss of DHg associated with water use activities, such as irrigation withdrawals, evaporation, and artificial wetland purification, is assumed to be constant at 20%. This assumption is based on water use statistics [10, 11], given the limited observational data on the mechanisms governing DHg loss.

In the box model, riverine Hg is simplified into two forms: particulate Hg and dissolved Hg. Methylmercury and other Hg species are not considered due to their relatively lower proportions. Hence, the below equations are applied:

$$PHg_{flux} + DHg_{flux} = THg_{flux} \quad \text{(e.S2)}$$

Where THg_{flux} is the total Hg flux in the global rivers (Mg/yr).

$$THg_{flux} \times Prop = PHg_{flux} \quad \text{(e.S3)}$$

Where the Prop is the *Proportion* of particulate phase Hg in global rivers versus total Hg (%).

Additionally, the inputs of riverine Hg are simplified into two components:

$$ErosionHg_{flux} + AnthHg_{flux} = THg_{flux} \quad \text{(e.S4)}$$

Where $ErosionHg_{flux}$ is the input erosional Hg flux of the global rivers (Mg/yr), and $AnthHg_{flux}$ is the input anthropogenic Hg flux of the global rivers (Mg/yr).

The Hg storage in the global reservoirs/dams is equal to the losses of the particulate phase Hg in the simplified box model:

$$HgStorage_{flux} = PHg_{flux} \times Rate_{dam\ trapping} / 12 \quad \text{(e.S5)}$$

Where $HgStorage_{flux}$ is the Hg accumulation rate in dams/reservoirs each month (Mg/mon).

To address uncertainties in the particulate phase Hg proportion in rivers, we established two scenarios: (1) 70%, based on observations from the Yangtze River and controlled experiments, representing the lower bound in global rivers, particularly in highly polluted regions; and (2) 90%, derived from summarized observation records, representing the upper bound (Table S1). Besides, the $ErosionHg_{flux}$ remains constant at approximately 420 Mg/yr and $Rate_{dam\ trapping}$ efficiency is ~48%, because those parameters are not influenced by the Hg forms (Fig. 1).

Firstly, we assume the $THg_{export\ flux}$ is fixed at approximately 1,000 Mg/yr. Under this condition, when the "proportion" is set to 70%, the total Hg flux (THg_{flux}) is ~1,656 Mg/yr, with the anthropogenic Hg flux ($AnthHg_{flux}$) at ~1,236 Mg/yr and a Hg accumulation rate of ~46 Mg/month. Increasing the "proportion" to 90% raises the THg_{flux} to ~1,825 Mg/yr, the $AnthHg_{flux}$ to ~1,405 Mg/yr, and the Hg accumulation rate to ~66 Mg/month. Alternatively, if we assume the THg export flux is adjustable while maintaining the AnthHgflux at ~1,500 Mg/yr, the results differ. With a "proportion" of 70%, the THg export flux reaches ~1,160 Mg/yr, and the Hg accumulation rate is ~54 Mg/month. Increasing the "proportion" to 90% results in a THg export flux of ~1,052 Mg/yr and a Hg accumulation rate of ~69 Mg/month. These results suggest that changes in the assumed Hg form have a relatively limited impact on model simulations.

In summary, our study concentrated on the particulate phase, which simplifies the Hg chemistry in the river channel and renders the model more feasible. However, we assert that this assumption is both reasonable and practical, provided that the global riverine Hg budget is predominantly dominated by the particulate phase, particularly in more polluted rivers. The relatively lower concentrations of dissolved Hg are anticipated to have minimal impact on the estimates of Hg settling and resuspension within riverine systems, encompassing channels and dams.

Text S2 Uncertainty analysis

The MOSART-Hg-WM model has uncertainties comparable to those in previous studies using the MOSART-Hg model [12]. We adopt the same model resolution as our previous study, which focuses on the pre-industrial era [12]. We find that the model with a higher resolution (0.5º×0.5º) performs better in simulating erosion flux in single topography units than a coarser resolution (1º×1º); however, the difference can be largely mitigated by using specific scaling factors. To balance computational cost and simulation accuracy, we use a resolution of 0.9º × 1.25º. In general, the accuracy of the erosion processes and riverine sediment/Hg delivery processes are validated against observations in previous studies [13, 14]. For instance, the model has better performance in simulating global sediment yield compared to alternative models such as the RUSLE model, with a 59% (mean) lower discrepancy relative to observations [13]. Additionally, sediment fluxes in the MOSART-Hg demonstrate similar performance to the BQART model [12].

The water management component (reservoirs/dams) accounts for sediment and riverine Hg trapping effects, alongside their influences on flow processes, which is supported by empirically validated relationships between reservoir properties and river parameters [15] (refer to Methods). The theoretical basin trapping by this method agrees well with the observed values, such as the Nile (observed 100% vs. theoretical 99%, mean), Kizil Irmak (observed 98% vs. theoretical 95%, mean) and Krishna River (observed 75% vs. theoretical 70%, mean) [15]. The water management module has also been validated independently in previous studies [11, 14]. Sediment flux estimations in MOSART-Hg-WM exhibit high performance compared to previous estimations [16] (Fig. S2). A similar level of model accuracy is expected for the Hg-trapping effects of reservoirs and dams.

Another source of uncertainties is the spatial distribution of anthropogenic Hg sources to freshwater environments. We consider two different atmospheric Hg inventories, AMAP inventory [17] and Streets inventory [18], as a proxy for the spatial distribution of anthropogenic releases to rivers. The riverine Hg export fluxes from both scenarios (*AMAP scenario* and *Streets scenario* hereafter) exhibit a strong correlation with observations at a global scale (Fig. S3). However, the different spatial distributions of Hg sources result in varied performances in different regions. The *Streets scenario* has a better performance in simulating riverine Hg in North America, while the *AMAP scenario* performs better in other regions, particularly due to the more detailed sector contributions for the latter.

The soil Hg concentration datasets play a crucial role in the Hg erosion processes. The diverse datasets (Wang dataset and Liu dataset, refer to Method) exert limited impacts on global-scale riverine Hg export fluxes, with the Liu dataset resulting in only a slight increase in riverine Hg export to oceans (<10%) than using the Wang dataset. Furthermore, the *Deposition Experiment* is devised to assess uncertainties arising from the change of soil Hg concentrations due to atmospheric Hg deposition and the loss of soil Hg by erosion (Table 1). In this experiment, soil Hg concentrations are dynamically adjusted based on the atmospheric deposition and erosion processes. However, the disparities between the scenarios with constant and dynamic soil Hg concentrations are minimal, with changes in riverine Hg export to oceans < 2% (Fig. S4). Similarly, given that the rates of gaseous Hg evaporating from rivers are lower than the atmospheric Hg deposition rates [19], the combined uncertainties from gaseous Hg evaporating and deposition are expected to remain within 2%.

The transformation of Hg forms in riverine processes is not explicitly accounted for in our model, and the assumption that all Hg exists in the particulate phase leads to an overestimation of both Hg trapping in reservoirs and anthropogenic Hg releases to rivers. Directly applying empirical particulate Hg fractions is unfeasible. Our macro-scale model cannot resolve dynamic local partitioning covariates like pH or DOC,

and extrapolating spatially biased observations would introduce unacceptable uncertainties. To address this uncertainty, we set up two scenarios for estimates: (1) 70% Particulate Hg: Based on observations from the Yangtze River and controlled ecosystem experiments, this scenario represents a lower bound; (2) 90% Particulate Hg: Based on observations from the Yukon River and prior estimates by Amos, et al. [5], this scenario represents an upper bound.

The results show that under the 70% and 90% particulate-phase scenarios, anthropogenic Hg releases are approximately 1,236 Mg/yr (mean) and 1,405 Mg/yr (mean), respectively, assuming a riverine Hg export of ~1,000 Mg/yr (mean) to oceans. Our estimated range of anthropogenic Hg releases (1,100–2,000 Mg/yr) effectively captures these uncertainties. The corresponding trapped Hg pools in reservoirs and dams are ~552 Mg/yr (mean) and ~792 Mg/yr (mean), respectively, which are lower than the ~900 Mg/yr (mean) estimated under the 100% particulate-phase assumption. Additionally, if anthropogenic Hg releases are held constant at ~1,500 Mg/yr, the riverine Hg export fluxes to oceans under the 70% and 90% particulate-phase scenarios are ~1,160 Mg/yr (mean) and ~1,052 Mg/yr (mean), respectively. Overall, the uncertainties arising from changes in Hg forms in our simulations have a limited impact on the global riverine Hg transport and export flux. Furthermore, the proportion of particulate-phase Hg in global rivers varies and is influenced by factors such as organic carbon content and pH [20]. Thus, incorporating biogeochemical cycling within an Earth system model can help improve riverine Hg estimates, however, which is currently absent in our approach. Details of these calculations are provided in Text S1.

Text S3 Implications for Hg cycling and sustainable science

Our assessments emphasize the role of dams/reservoirs as the sink in global Hg biogeochemistry. The accumulation of Hg in global reservoirs adds complexity to environmental governance efforts. In the short term, Hg pools increase through particulate sedimentation, while transformations such as changes in Hg speciation and bioavailability can reduce the mobile fraction. Despite high Hg levels, reservoir trapping efficiency remains largely unaffected, as it is driven primarily by sediment accumulation rather than Hg content. Over the long term in some reservoirs, Hg buried in deeper sediment layers, with limited exchange with bottom waters and have no possibility to be resuspended, can be considered effectively sequestered, particularly in deeper or smaller reservoirs, provided dredging does not occur. Management actions such as dredging can help remove sediment-bound Hg from the riverbed, reducing its potential remobilization into the aquatic system. The settled Hg in reservoirs creates a suitable environment for Hg methylation [21], with conditions similar to those of nutrient-rich wetlands that support biological activity and methylation.

Our findings provide insights into the size and behavior of Hg pools in global reservoirs, which can be used to assess methylation risks for human health and inform future monitoring efforts. Hg pollution has long been recognized as a sustainability challenge [22], particularly because riverine Hg contamination poses direct threats to multiple the United Nations Sustainable Development Goals (SDGs) (https://www.undp.org/sustainable-development-goals)—notably SDG 3 (Good Health and Well-being) and SDG 6 (Clean Water and Sanitation). While water infrastructure supports agriculture, hydropower, and flood control, Hg accumulated in reservoir sediments and riverbeds can serve as secondary sources of contamination [23, 24], posing risks to both food and water security. For example, observations from the Yangtze River suggest that Hg retained in the Three Gorges Reservoir has become a downstream source of riverine Hg due to declining upstream Hg inputs [24]. Watershed management practices, such as dam construction, hydroelectric development, and water-sediment regulation, can significantly influence Hg fluxes and deposition patterns. Hydropower reservoirs may create conditions conducive to methylation [25],

leading to elevated methylmercury concentrations in rivers, estuaries, and aquatic organisms [26]; while sediment flushing operations alter the downstream transport and fate of particulate-bound Hg [27].

Our model provides a process-based, spatially explicit scientific framework for assessing and managing the risks associated with Hg pollution in river systems. It supports the development of targeted mitigation strategies to reduce human exposure and promote more sustainable water infrastructure planning. In particular, our global estimates of Hg accumulation in reservoirs can inform environmental restoration efforts, enhance monitoring under international frameworks such as the Minamata Convention on Mercury, and contribute to SDG 17 (Partnerships for the Goals) (https://www.undp.org/sustainable-development-goals) by facilitating data-driven collaboration across regions. This work also offers a science-based foundation for risk assessments related to dam operation, modification, and removal, supporting integrated watershed and infrastructure management.

Figures

Figure S1 Seasonal fluxes of riverine mercury discharge over land

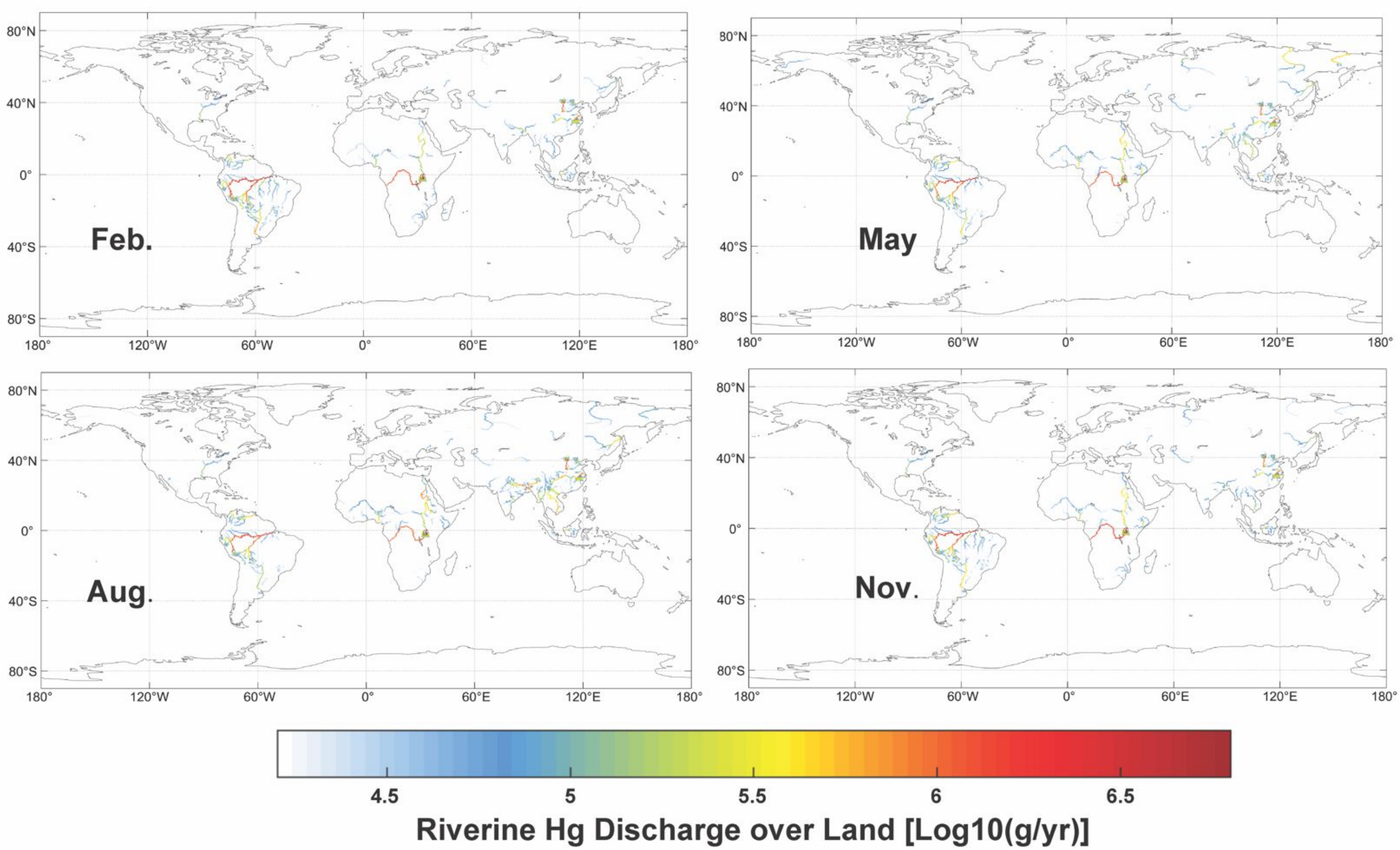

Figure S2 The sediment load of estimation versus other estimations [16]. The 1950/2010 sediment load is referred to as the estimation by Syvitski, et al. [16].

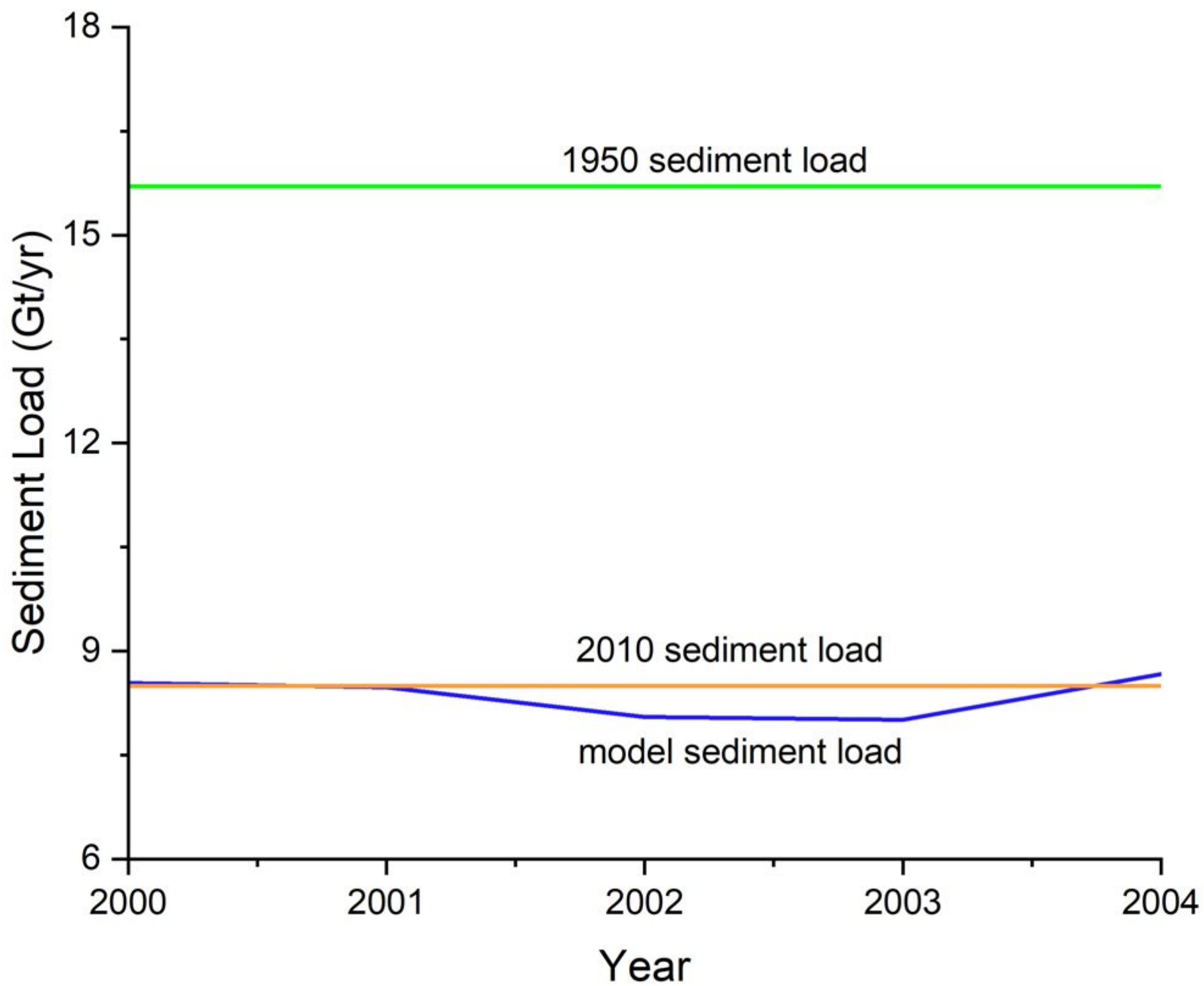

Figure S3 The comparison of modeled riverine Hg fluxes export by rivers under Inventory Experiment with observation, AMAP represents the AMAP inventory, the Streets represent the Streets inventory (refer to Table 1). Unit kg/yr.

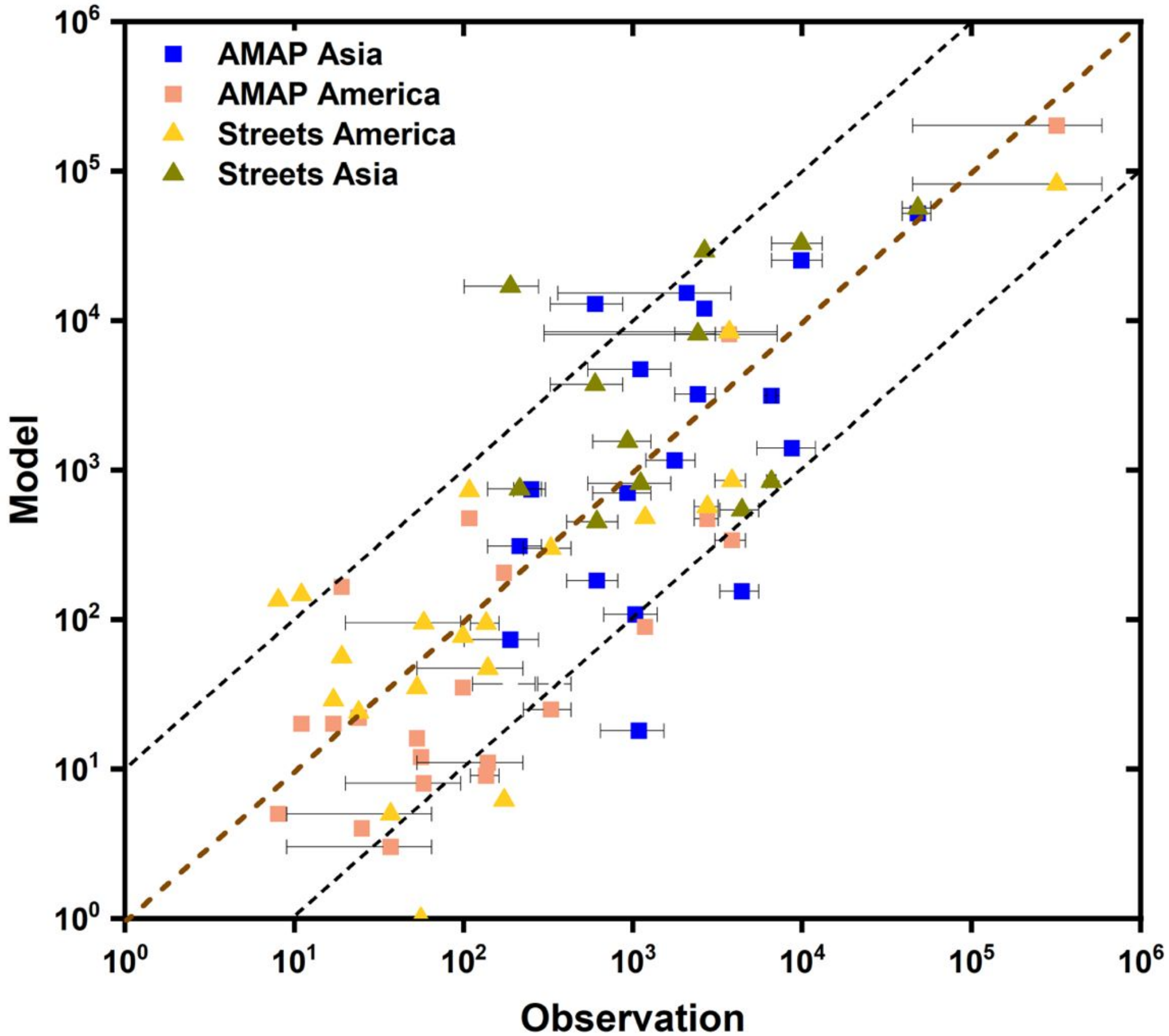

Figure S4 The comparison of Model results by *Soil Hg Experiment* with observation, Wang represents the soil Hg concentration of Wang, et al. [28], the Chen represents the soil Hg concentration dataset of Liu, et al. [29] (refer to Table 1).

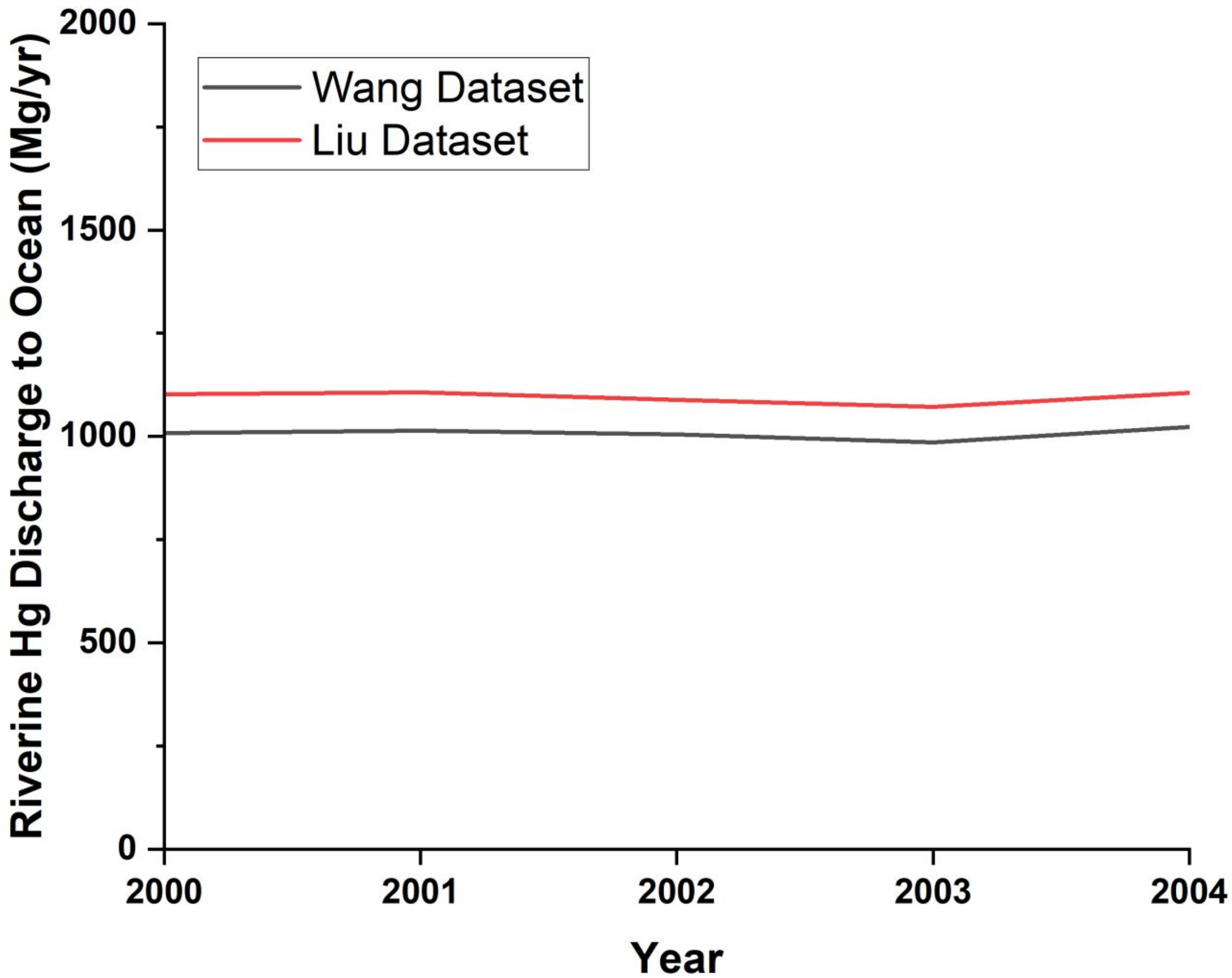

Figure S5 The comparison of Model results by *Deposition Experiment*, the Deposition represents the condition with dynamic soil Hg and atmospheric Hg deposition in Table 1, and the Without Deposition represents the condition with fixed soil Hg concentration in Table 1.

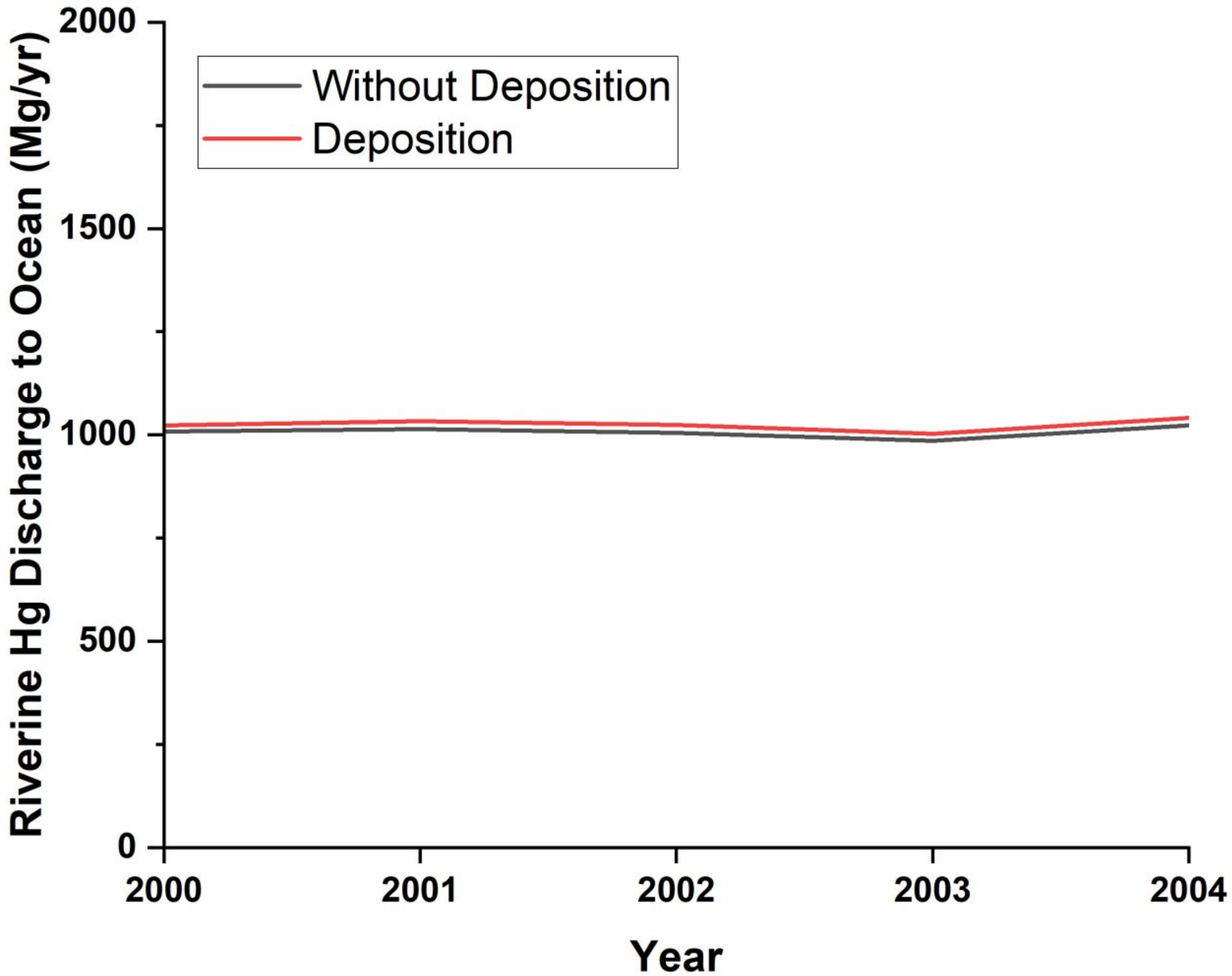

Tables

Table S1 Hg Forms in Global Rivers and the Proportion of Particulate Phase Hg Relative to Total Hg (Parts of the records are summary based on Supplementary Table 1 in Liu, et al. [3])

River/Place Name	Region	Total Hg concentration, ng/L	Particulate Hg concentration, ng/L	Dissolved Hg concentration, ng/L	Proportion of particulate Hg/total Hg, %	References
Amazon's main tributaries (rising water stage)	Americas	5	2	3	40%	20
Amazon's main tributaries (dry season)		4.2	1	3.2	24%	
Amazon main tributaries (rising water stage)		28.2	25.6	2.6	91%	
Amazon main tributaries (flood peak)		18.8	17	1.8	90%	
Amazon's main tributaries (dry season)		10	7.8	2.2	78%	
Amazon main tributaries (rising water stage)		37.1	33.1	4	89%	
Amazon main tributaries (flood peak)		14.6	11.8	2.8	81%	
Amazon's main tributaries (dry season)		7.4	5.6	1.8	76%	
Amazon River (rising water stage)		19.1	15.7	3.4	82%	
Amazon River (flood peak)		6.4	1.8	4.6	28%	
Amazon River (dry season)		33.4	31	2.4	93%	
Marano Lagoon (Italy)	Europe	44	32	13	73%	30
Austria Sites		0.37	0.16	0.21	43%	31
Austria Sites		0.57	0.39	0.18	68%	
Austria Sites		0.95	0.13	0.82	14%	
Bei River (China)	Asia	5.6	4.6	1	82%	3
Bei River (China)		8.4	6.5	1.9	77%	
Bei River (China)		9.9	8.7	1.2	88%	

Bei River (China)		12	10	1.6	83%	
Berry Creek Estuary	Americas	280	240	40	86%	32
Bulgaria Sites	Europe	0.26	0.02	0.24	8%	31
Bulgaria Sites		0.28	0.07	0.21	25%	
Churchill	Americas	2	0.3	1.7	15%	1
Churchill		2	0.3	1.7	15%	33
Churchill		3.5	0.2	3.3	6%	34
Connecticut		2.2	0.96	1.2	44%	35
Connecticut		2.2	1.5	0.75	68%	
Connecticut		2.4	0.81	1.6	34%	
Connecticut		2.8	1.1	1.8	39%	
Connecticut		3.2	1.6	1.6	50%	
Connecticut		3.4	1.5	1.9	44%	
Connecticut		3.5	2.5	0.97	71%	
Connecticut		4.3	0.96	3.4	22%	
Connecticut		4.4	2.7	1.7	61%	
Connecticut		4.7	4	0.73	85%	
Connecticut		4.9	1.8	3.1	37%	
Connecticut		5	3.1	2	62%	
Connecticut		5.3	1.9	3.4	36%	
Connecticut		9.3	6	3.3	65%	
Corno	Europe	40	16	24	40%	30
Dong River (China)	Asia	6.1	3.7	2.4	61%	3
Dong River (China)		6.1	4.3	1.8	70%	
Dong River (China)		9.2	6.6	2.6	72%	
Dong River (China)		12	10	1.8	83%	
East River (China)		17	5.9	12	35%	36
East River (China)		17	8	9	47%	

East River (China)		17	11	6.3	65%	
East River (China)		18	5.5	13	31%	
East River (China)		19	8.3	11	44%	
East River (China)		19	11	7.9	58%	
East River (China)		21	6.9	14	33%	
East River (China)		24	8	16	33%	
East River (China)		24	8.2	15	34%	
France Sites	Europe	0.51	0.03	0.48	6%	31
France Sites		0.88	0.47	0.41	53%	
Garonne River La Réole		11	9.9	0.6	90%	37
Garonne River La Réole		15	14	1.2	93%	
Garonne River La Réole		19	18	0.9	95%	
Garonne River La Réole		23	22	0.7	96%	
Garonne River La Réole		40	39	1	98%	
Garonne River P.-Ste-Marie		8	7.3	0.7	91%	
Garonne River P.-Ste-Marie		12	11	1.2	92%	
Garonne River P.-Ste-Marie		14	13	0.9	93%	
Great Whale River	Americas	2.8	0.6	2.2	21%	34
Great Britain Sites	Europe	0.28	0.12	0.16	43%	31
Great Britain Sites		0.42	0.18	0.24	43%	
Great Britain Sites		2.8	0.48	2.3	17%	
Guandu River	Americas	220	190	35	86%	38
Guarda River		20	19	0.5	95%	
Hackensack River		16	14	2.1	88%	32
Han River	Asia	14	4.3	10	31%	3
Han River		23	5.7	18	25%	
Huai River		53	43	10	81%	39
Huangpu River		15	11	4.3	73%	3

Huangpu River		160	150	4.5	94%	
Hudson River		13	12	0.62	92%	40
Ita River	Americas	1.6	0.27	1.4	17%	
Itimirim River		1	0.4	0.4	40%	38
Itinguçu River		2.4	1.2	1.2	50%	
Jin River (China)		7	3.8	3.2	54%	
Jin River (China)		8	4.6	3.4	58%	
Jiulong River (China)		8.6	4.5	4.1	52%	3
Jiulong River (China)		10	6.3	3.8	63%	
Lena River		1.7	1	0.7	59%	
Lena River		1.8	1.1	0.64	61%	41
Lena River		1.9	0.86	1.1	45%	
Lena River		1.9	1.3	0.6	68%	
Lena River	Asia	2.6	1.6	1	62%	41; 1
Lena River		2.9	2.4	0.5	83%	41
Lena River		4.8	3.9	0.9	81%	
Liao River		11	7.8	3.5	71%	
Liao River		12	8.6	3.3	72%	
Liao River		13	11	2.2	85%	3
Liao River		14	10	4.4	71%	
Luan River		12	8.4	3.8	70%	
Luan River		13	9.1	3.7	70%	
Mackenzie River		7	4.2	2.8	60%	2
Mackenzie River		15	13	1.6	87%	42
Maderia River	Americas	9.5	3.9	5.5	41%	43
Major tributaries flowing into the Gulf of Maine		4.4	2.2	2.2	50%	44
Mekong River	Asia	0.92	0.52	0.4	57%	45
Mekong River		0.92	0.68	0.24	74%	

Mekong River		0.94	0.7	0.24	74%	
Mekong River		1.5	1.2	0.32	80%	
Mekong River		2.2	1.3	0.88	59%	
Mekong River		2.4	1.5	0.9	63%	
Mekong River		2.4	1.8	0.62	75%	
Mekong River		3.4	2.3	1.1	68%	
Mekong River		5.2	4.6	0.62	88%	
Mekong River		6.4	5.4	0.96	84%	
Mekong River		6.8	5.2	1.6	76%	
Mekong River		7.8	6.5	1.3	83%	
Mekong River		9	7.4	1.6	82%	
Mekong River		11	8	2.8	73%	
Mekong River		11	10	0.56	91%	
Mekong River		16	14	2	88%	
Min River		10	7	3.3	70%	3
Min River		11	8.1	3	74%	
Min River		12	7.1	4.9	59%	
Min River		17	10	6.8	59%	
Mississippi River	Americas	5.8	4.7	1.1	81%	1
Nandu River	Asia	8.5	4.6	3.8	54%	3
Nandu River		11	6.5	4.2	59%	
Negro River	Americas	12	5.6	6	47%	43
Negro River		18	11	7.1	61%	
Nelson River		0.88	0.38	0.5	43%	33
Nelson River		0.9	0.4	0.5	44%	1
Nelson River		2.2	0.3	1.9	14%	34
New York/New Jersey Harbor Estuary		17	16	0.62	94%	40
Nile River	Asia	2.2	1.8	0.36	82%	46

Ob' River		0.56	0.14	0.42	25%	41
Ob' River		0.62	0.22	0.4	35%	
Ob' River		0.84	0.26	0.58	31%	
Ob' River		0.97	0.21	0.76	22%	
Ob' River		1.1	0.52	0.62	47%	
Ob' River		1.2	0.58	0.58	48%	
Ob' River		1.3	0.65	0.62	50%	
Ob' River		1.7	1.1	0.6	65%	41; 1
Ob' River		2.5	2	0.48	80%	41
Ob' River		7.4	6.8	0.64	92%	
Ou River		10	6.4	3.7	64%	3
Ou River		14	11	3.4	79%	
Peel River	Americas	22	20	2	91%	42
Qiantang River	Asia	8.9	6.7	2.2	75%	3
Qiantang River		12	8.4	3.8	70%	
Qiantang River		16	12	4.2	75%	
Qiantang River		19	15	4.1	79%	
Rhône River	Europe	6	4.9	1.1	82%	46
San Francisco Estuary	Americas	6	4.8	1.2	80%	47
San Francisco Estuary		6.6	5.2	1.4	79%	48
São Francisco River		37	37	0.25	100%	38
Sinnamary estuary		22	21	1.2	95%	49
Solimoes		7.2	1.9	5.3	26%	43
Solimoes		9.2	3	6.2	33%	
Solimoes		11	5.9	5.5	54%	
Solimoes		14	7	6.9	50%	
Spain Sites	Europe	0.37	0.09	0.28	24%	31
Spain Sites		0.46	0.09	0.37	20%	

Spain Sites		0.94	0.36	0.58	38%	
St. Lawrence River	Americas	2.8	2.2	0.6	79%	1
Sweden Sites	Europe	0.18	0.02	0.16	11%	31
Sweden Sites		0.86	0.25	0.61	29%	
Xi River	Asia	4.5	3.4	1.2	76%	3
Xi River		6	2.4	3.6	40%	
Xi River		11	7.9	2.6	72%	
Xi River		24	20	4.2	83%	
Yalu River		10	6.8	3.5	68%	
Yalu River		14	11	3.2	79%	
Yalu River		57	37	20	65%	50
Yangtze River		35	24	11	69%	51
Yangtze River		17	11	5.8	65%	23
Yangtze River		19	8.2	11	43%	
Yangtze River		19	13	6.5	68%	
Yangtze River		20	12	7.9	60%	
Yangtze River		22	8.7	14	40%	
Yangtze River		22	9.9	12	45%	
Yangtze River		25	12	13	48%	
Yangtze River		29	17	11	59%	
Yangtze River		30	18	11	60%	
Yangtze River		33	19	14	58%	
Yangtze River		34	16	18	47%	
Yangtze River		39	20	19	51%	
Yangtze River		93	69	24	74%	
Yellow River		19	17	1.9	89%	3
Yellow River		25	21	4	84%	
Yellow River		27	20	6.4	74%	

Yellow River		29	25	3.9	86%	
Yenisei River		0.35	0.19	0.16	54%	41
Yenisei River		0.35	0.21	0.14	60%	
Yenisei River		0.45	0.29	0.16	64%	
Yenisei River		0.51	0.31	0.2	61%	
Yenisei River		0.58	0.24	0.34	41%	
Yenisei River		0.7	0.28	0.42	40%	
Yenisei River		0.71	0.27	0.44	38%	
Yenisei River		0.8	0.5	0.3	63%	1, 41
Yenisei River		0.82	0.14	0.68	17%	41
Yenisei River		0.82	0.54	0.28	66%	
Yenisei River		0.95	0.39	0.56	41%	
Yenisei River		0.96	0.72	0.24	75%	
Yenisei River		1.5	1.2	0.3	80%	
Yenisei River		2.7	1.8	0.88	67%	
Yukon River	Americas	15	13	1.9	87%	1

References

(1) Schuster, P. F.; Striegl, R. G.; Aiken, G. R.; Krabbenhoft, D. P.; Dewild, J. F.; Butler, K.; Kamark, B.; Dornblaser, M. Mercury export from the Yukon River Basin and potential response to a changing climate. *Environmental Science & Technology* **2011**, *45* (21), 9262-9267. DOI: 10.1021/es202068b.

(2) Leitch, D. R.; Carrie, J.; Lean, D.; Macdonald, R. W.; Stern, G. A.; Wang, F. The delivery of mercury to the Beaufort Sea of the Arctic Ocean by the Mackenzie River. *Science of The Total Environment* **2007**, *373* (1), 178-195. DOI: 10.1016/j.scitotenv.2006.10.041.

(3) Liu, M.; Zhang, Q.; Maavara, T.; Liu, S.; Wang, X.; Raymond, P. A. Rivers as the largest source of mercury to coastal oceans worldwide. *Nature Geoscience* **2021**, *14* (9), 672-677. DOI: 10.1038/s41561-021-00793-2.

(4) Sonke, J. E.; Teisserenc, R.; Heimbürger-Boavida, L.-E.; Petrova, M. V.; Marusczak, N.; Le Dantec, T.; Chupakov, A. V.; Li, C.; Thackray, C. P.; Sunderland, E. M.; et al. Eurasian river spring flood observations support net Arctic Ocean mercury export to the atmosphere and Atlantic Ocean. *Proceedings of the National Academy of Sciences* **2018**, *115* (50). DOI: 10.1073/pnas.1811957115.

(5) Amos, H. M.; Jacob, D. J.; Kocman, D.; Horowitz, H. M.; Zhang, Y.; Dutkiewicz, S.; Horvat, M.; Corbitt, E. S.; Krabbenhoft, D. P.; Sunderland, E. M. Global biogeochemical implications of mercury discharges from rivers and sediment burial. *Environmental Science and Technology* **2014**, *48* (16), 9514-9522. DOI: 10.1021/es502134t.

(6) Ram, A.; Rokade, M. A.; Borole, D. V.; Zingde, M. D. Mercury in sediments of Ulhas estuary. *Marine Pollution Bulletin* **2003**, *46* (7), 846-857. DOI: 10.1016/s0025-326x(03)00065-1.

(7) Sankar, R.; Ramkumar, L.; Rajkumar, M.; Sun, J.; Ananthan, G. Seasonal variations in physico-chemical parameters and heavy metals in water and sediments of Uppanar estuary, Nagapattinam, India. *J Environ Biol* **2010**, *31* (5), 681-686.

(8) Kocman, D.; Kanduč, T.; Ogrinc, N.; Horvat, M. Distribution and partitioning of mercury in a river catchment impacted by former mercury mining activity. *Biogeochemistry* **2010**, *104* (1-3), 183-201. DOI: 10.1007/s10533-010-9495-5.

(9) Lu, X.; Johnson, K. W.; Whitney, F. A.; Wong, C. S.; Wu, J. A study on the flux, speciation, and budget of mercury in controlled experimental ecosystems. *Chinese Journal of Oceanology and Limnology* **1987**, *5* (2), 146-160. DOI: 10.1007/bf02844999.

(10) McDermid, S.; Nocco, M.; Lawston-Parker, P.; Keune, J.; Pokhrel, Y.; Jain, M.; Jägermeyr, J.; Brocca, L.; Massari, C.; Jones, A. D.; et al. Irrigation in the Earth system. *Nature Reviews Earth & Environment* **2023**, *4* (7), 435-453. DOI: 10.1038/s43017-023-00438-5.

(11) Zhou, T.; Leung, L. R.; Leng, G.; Voisin, N.; Li, H. Y.; Craig, A. P.; Tesfa, T.; Mao, Y. Global irrigation characteristics and effects simulated by fully coupled land surface, river, and water management models in E3SM. *J Adv Model Earth Sy* **2020**, *12* (10). DOI: 10.1029/2020ms002069.

(12) Peng, D.; Tan, Z.; Yuan, T.; Wu, P.; Song, Z.; Zhang, P.; Huang, S.; Zhang, Y.; Lei, T.; Middleton, B. A.; et al. Human perturbations to mercury in global rivers. *Sci Adv* **2025**, *11* (24), eadw0471. DOI: 10.1126/sciadv.adw0471 From NLM Medline.

(13) Tan, Z. L.; Leung, L. R.; Li, H. Y.; Tesfa, T. Modeling sediment yield in land surface and earth system models: model comparison, development, and evaluation. *J Adv Model Earth Sy* **2018**, *10* (9), 2192-2213, Article. DOI: 10.1029/2017ms001270.

(14) Li, H.-Y.; Tan, Z.; Ma, H.; Zhu, Z.; Abeshu, G. W.; Zhu, S.; Cohen, S.; Zhou, T.; Xu, D.; Leung, L. R. A new large-scale suspended sediment model and its application over the United States. *Hydrology and Earth System Sciences* **2022**, *26* (3), 665-688. DOI: 10.5194/hess-26-665-2022.

(15) Vörösmarty, C. J.; Meybeck, M.; Fekete, B.; Sharma, K.; Green, P.; Syvitski, J. P. M. Anthropogenic

sediment retention: major global impact from registered river impoundments. *Global and Planetary Change* **2003**, *39* (1-2), 169-190. DOI: 10.1016/s0921-8181(03)00023-7.
(16) Syvitski, J.; Ángel, J. R.; Saito, Y.; Overeem, I.; Vörösmarty, C. J.; Wang, H.; Olago, D. Earth's sediment cycle during the Anthropocene. *Nature Reviews Earth & Environment* **2022**, *3* (3), 179-196. DOI: 10.1038/s43017-021-00253-w.
(17) Steenhuisen, F.; Wilson, S. J. Geospatially distributed (gridded) global mercury emissions to air from anthropogenic sources in 2015. *DataverseNL* **2022**. DOI: doi:10.34894/SZ2KOI. Steenhuisen, F.; Wilson, S. J. Development and application of an updated geospatial distribution model for gridding 2015 global mercury emissions. *Atmos. Environ.* **2019**, *211*, 138-150. DOI: 10.1016/j.atmosenv.2019.05.003.
(18) Streets, D. G.; Horowitz, H. M.; Lu, Z.; Levin, L.; Thackray, C. P.; Sunderland, E. M. Global and regional trends in mercury emissions and concentrations, 2010–2015. *Atmos. Environ.* **2019**, *201*, 417-427. DOI: 10.1016/j.atmosenv.2018.12.031.
(19) da Silva, G. S.; Jardim, W. F.; Fadini, P. S. Elemental gaseous mercury flux at the water/air interface over the Negro River basin, Amazon, Brazil. *Science of The Total Environment* **2006**, *368* (1), 189-198. DOI: 10.1016/j.scitotenv.2005.09.082.
(20) Maia, P. D.; Maurice, L.; Tessier, E.; Amouroux, D.; Cossa, D.; Pérez, M.; Moreira-Turcq, P.; Rhéault, I. Mercury distribution and exchanges between the Amazon River and connected floodplain lakes. *Science of The Total Environment* **2009**, *407* (23), 6073-6084. DOI: 10.1016/j.scitotenv.2009.08.015.
(21) Hsu-Kim, H.; Eckley, C. S.; Achá, D.; Feng, X.; Gilmour, C. C.; Jonsson, S.; Mitchell, C. P. J. Challenges and opportunities for managing aquatic mercury pollution in altered landscapes. *Ambio* **2018**, *47* (2), 141-169. DOI: 10.1007/s13280-017-1006-7. Ma, H.; Chen, Q.; Liu, D.; Chen, Y.; Lin, Y.; Zhang, J.; Yan, X. Impacts of river damming on sediment methylmercury dynamics. *ACS ES&T Water* **2023**, *3* (4), 934-942. DOI: 10.1021/acsestwater.2c00397. Pestana, I. A.; Azevedo, L. S.; Bastos, W. R.; Magalhães de Souza, C. M. The impact of hydroelectric dams on mercury dynamics in South America: A review. *Chemosphere* **2019**, *219*, 546-556. DOI: 10.1016/j.chemosphere.2018.12.035.
(22) Sodeno, R. Analysis of the Minamata Convention on Mercury in the Context of Sustainable Development Goals (SDGs). *Global Environmental Research* **2020**, 065-070. Selin, H.; Selin, N. E. From Stockholm to Minamata and beyond: Governing mercury pollution for a more sustainable future. *One Earth* **2022**, *5* (10), 1109-1125. DOI: 10.1016/j.oneear.2022.09.001.
(23) Liu, M.; He, Y.; Baumann, Z.; Zhang, Q.; Jing, X.; Mason, R. P.; Xie, H.; Shen, H.; Chen, L.; Zhang, W.; et al. The impact of the Three Gorges Dam on the fate of metal contaminants across the river-ocean continuum. *Water Research* **2020**, *185*, 116295. DOI: 10.1016/j.watres.2020.116295.
(24) Peng, D.; Lyu, J.; Song, Z.; Huang, S.; Zhang, P.; Gao, J.; Zhang, Y. Mercury budgets in the suspended particulate matters of the Yangtze River. *Water Research* **2023**, *243*. DOI: 10.1016/j.watres.2023.120390.
(25) Amyot, M.; Bilodeau, F.; Tremblay, A.; Planas, D.; Walsh, D.; Ponton, D. E. Cumulative effects of watershed disturbances and run-of-river dams on mercury cycling: case study and recommendations for environmental managers. *Environmental Management* **2024**. DOI: 10.1007/s00267-024-01990-6.
(26) Calder, R. S. D.; Schartup, A. T.; Li, M.; Valberg, A. P.; Balcom, P. H.; Sunderland, E. M. Future Impacts of Hydroelectric Power Development on Methylmercury Exposures of Canadian Indigenous Communities. *Environmental Science & Technology* **2016**, *50* (23), 13115-13122. DOI: 10.1021/acs.est.6b04447.
(27) Che, X.; Liu, M.; Sun, X.; Chen, J.; Ding, Y.; Fan, D. Transport of particulate mercury along the reservoir-downstream - estuary continuum during the water-sediment regulation scheme period of Yellow River in 2018. *Journal of Hazardous Materials* **2025**, *485*. DOI: 10.1016/j.jhazmat.2024.136916.
(28) Wang, X.; Yuan, W.; Lin, C.-J.; Zhang, L.; Zhang, H.; Feng, X. Climate and vegetation as primary drivers

for global mercury storage in surface soil. *Environmental Science & Technology* **2019**, *53* (18), 10665-10675. DOI: 10.1021/acs.est.9b02386.

(29) Liu, Y.-R.; Guo, L.; Yang, Z.; Xu, Z.; Zhao, J.; Wen, S.-H.; Delgado-Baquerizo, M.; Chen, L. Multidimensional drivers of mercury distribution in global surface soils: insights from a global standardized field survey. *Environmental Science & Technology* **2023**, *57* (33), 12442-12452. DOI: 10.1021/acs.est.3c04313.

(30) Covelli, S.; Acquavita, A.; Piani, R.; Predonzani, S.; De Vittor, C. Recent contamination of mercury in an estuarine environment (Marano lagoon, Northern Adriatic, Italy). *Estuarine, Coastal and Shelf Science* **2009**, *82* (2), 273-284. DOI: 10.1016/j.ecss.2009.01.021.

(31) Bravo, A. G.; Kothawala, D. N.; Attermeyer, K.; Tessier, E.; Bodmer, P.; Ledesma, J. L. J.; Audet, J.; Casas-Ruiz, J. P.; Catalán, N.; Cauvy-Fraunié, S.; et al. The interplay between total mercury, methylmercury and dissolved organic matter in fluvial systems: A latitudinal study across Europe. *Water Research* **2018**, *144*, 172-182. DOI: 10.1016/j.watres.2018.06.064.

(32) Cardona-Marek, T.; Schaefer, J.; Ellickson, K.; Barkay, T.; Reinfelder, J. R. Mercury Speciation, Reactivity, and Bioavailability in a Highly Contaminated Estuary, Berry's Creek, New Jersey Meadowlands. *Environmental Science & Technology* **2007**, *41* (24), 8268-8274. DOI: 10.1021/es070945h.

(33) Kirk, J. L.; St. Louis, V. L.; Hintelmann, H.; Lehnherr, I.; Else, B.; Poissant, L. Methylated Mercury Species in Marine Waters of the Canadian High and Sub Arctic. *Environmental Science & Technology* **2008**, *42* (22), 8367-8373. DOI: 10.1021/es801635m.

(34) Hare, A.; Stern, G. A.; Macdonald, R. W.; Kuzyk, Z. Z.; Wang, F. Contemporary and preindustrial mass budgets of mercury in the Hudson Bay Marine System: The role of sediment recycling. *Science of The Total Environment* **2008**, *406* (1-2), 190-204. DOI: 10.1016/j.scitotenv.2008.07.033.

(35) Balcom, P. H.; Fitzgerald, W. F.; Vandal, G. M.; Lamborg, C. H.; Rolfhus, K. R.; Langer, C. S.; Hammerschmidt, C. R. Mercury sources and cycling in the Connecticut River and Long Island Sound. *Marine Chemistry* **2004**, *90* (1-4), 53-74. DOI: 10.1016/j.marchem.2004.02.020.

(36) Liu, J.; Feng, X.; Zhu, W.; Zhang, X.; Yin, R. Spatial distribution and speciation of mercury and methyl mercury in the surface water of East River (Dongjiang) tributary of Pearl River Delta, South China. *Environ. Sci. Pollut. Res.* **2011**, *19* (1), 105-112. DOI: 10.1007/s11356-011-0542-0.

(37) Schäfer, J.; Blanc, G.; Audry, S.; Cossa, D.; Bossy, C. Mercury in the Lot–Garonne River system (France): Sources, fluxes and anthropogenic component. *Applied Geochemistry* **2006**, *21* (3), 515-527. DOI: 10.1016/j.apgeochem.2005.12.004.

(38) Paraquetti, H. H. M.; Ayres, G. A.; de Almeida, M. D.; Molisani, M. M.; Lacerda, L. D. d. Mercury distribution, speciation and flux in the Sepetiba Bay tributaries, SE Brazil. *Water Research* **2004**, *38* (6), 1439-1448. DOI: 10.1016/j.watres.2003.11.039.

(39) Luo Bin, L. B.; Liu Ling, L. L.; Zhang JinLiang , Z. J. Levels and distribution characteristics of heavy metals in sediments in main stream of Huaihe River. *Journal of Environment and Health* **2010**, *27* (12), 1122–1127.

(40) Balcom, P. H.; Hammerschmidt, C. R.; Fitzgerald, W. F.; Lamborg, C. H.; O'Connor, J. S. Seasonal distributions and cycling of mercury and methylmercury in the waters of New York/New Jersey Harbor Estuary. *Marine Chemistry* **2008**, *109* (1-2), 1-17. DOI: 10.1016/j.marchem.2007.09.005.

(41) Coquery, M.; Cossa, D. Mercury speciation in surface waters of the north sea. *Netherlands Journal of Sea Research* **1995**, *34* (4), 245-257. DOI: 10.1016/0077-7579(95)90035-7.

(42) Emmerton, C. A.; Graydon, J. A.; Gareis, J. A. L.; St. Louis, V. L.; Lesack, L. F. W.; Banack, J. K. A.; Hicks, F.; Nafziger, J. Mercury export to the arctic ocean from the Mackenzie River, Canada. *Environmental Science & Technology* **2013**, *47* (14), 7644-7654. DOI: 10.1021/es400715r.

(43) Maurice-Bourgoin, L.; Quemerais, B.; Moreira-Turcq, P.; Seyler, P. Transport, distribution and speciation of

mercury in the Amazon River at the confluence of black and white waters of the Negro and Solimões Rivers. *Hydrological Processes* **2003**, *17* (7), 1405-1417. DOI: 10.1002/hyp.1292.
(44) Sunderland, E. M.; Mason, R. P. Human impacts on open ocean mercury concentrations. *Global Biogeochem Cy* **2007**, *21* (4). DOI: 10.1029/2006gb002876.
(45) Noh, S.; Choi, M.; Kim, E.; Dan, N. P.; Thanh, B. X.; Ha, N. T. V.; Sthiannopkao, S.; Han, S. Influence of salinity intrusion on the speciation and partitioning of mercury in the Mekong River Delta. *Geochimica et Cosmochimica Acta* **2013**, *106*, 379-390. DOI: 10.1016/j.gca.2012.12.018.
(46) Cossa, D.; Coquery, M.; Gobeil, C.; Martin, J. M. Mercury Fluxes at the Ocean Margins. In *Global and Regional Mercury Cycles: Sources, Fluxes and Mass Balances*, 1996; pp 229-247.
(47) Conaway, C. H.; Black, F. J.; Grieb, T. M.; Roy, S.; Flegal, A. R. Mercury in the San Francisco estuary. *Reviews of environmental contamination and toxicology* **2008**, 29-54.
(48) Bergamaschi, B. A.; Fleck, J. A.; Downing, B. D.; Boss, E.; Pellerin, B. A.; Ganju, N. K.; Schoellhamer, D. H.; Byington, A. A.; Heim, W. A.; Stephenson, M.; et al. Mercury Dynamics in a San Francisco Estuary Tidal Wetland: Assessing Dynamics Using In Situ Measurements. *Estuaries and Coasts* **2012**, *35* (4), 1036-1048. DOI: 10.1007/s12237-012-9501-3.
(49) Muresan, B.; Cossa, D.; Coquery, M.; Richard, S. Mercury sources and transformations in a man-perturbed tidal estuary: The Sinnamary Estuary, French Guiana. *Geochimica et Cosmochimica Acta* **2008**, *72* (22), 5416-5430. DOI: 10.1016/j.gca.2008.08.021.
(50) Liu, S.; Zhang, J.; Cui, J. Mercury in four north China estuaries: the Daliaohe, Yalujiang, Luanhe and Dongcunhe. *Journal of Ocean University of Qingdao/Qingdao Haiyang Daxue Xuebao. Qingdao* **2001**, *31* (1), 136-142.
(51) Müller, B.; Berg, M.; Yao, Z. P.; Zhang, X. F.; Wang, D.; Pfluger, A. How polluted is the Yangtze river? Water quality downstream from the Three Gorges Dam. *Science of the total environment* **2008**, *402* (2-3), 232-247.
